# Functional Screening Identifies miRNAs Influencing Apoptosis and Proliferation in Colorectal Cancer

**DOI:** 10.1371/journal.pone.0096767

**Published:** 2014-06-03

**Authors:** Lise Lotte Christensen, Anja Holm, Juha Rantala, Olli Kallioniemi, Mads H. Rasmussen, Marie S. Ostenfeld, Frederik Dagnaes-Hansen, Bodil Øster, Troels Schepeler, Heidi Tobiasen, Kasper Thorsen, Oliver M. Sieber, Peter Gibbs, Philippe Lamy, Torben F. Hansen, Anders Jakobsen, Eva M. Riising, Kristian Helin, Jan Lubinski, Rikke Hagemann-Madsen, Søren Laurberg, Torben F. Ørntoft, Claus L. Andersen

**Affiliations:** 1 Colorectal Cancer Research Group, Department of Molecular Medicine (MOMA), Aarhus University Hospital, University of Aarhus, Aarhus, Denmark; 2 Clinical Biochemistry, Glostrup Research Institute, Glostrup Hospital, Glostrup, Denmark; 3 Molecular Sleep Laboratory, Department of Diagnostics, and Danish Center for Sleep Medicine, Department of Clinical Neurophysiology, Glostrup Hospital, University of Copenhagen, Glostrup, Denmark; 4 Medical Biotechnology, VTT Technical Research Centre of Finland, Turku, Finland; 5 Bladder Cancer Research Group, Department of Molecular Medicine (MOMA), Aarhus University Hospital, University of Aarhus, Aarhus, Denmark; 6 Department of Biomedicine, University of Aarhus, Aarhus, Denmark; 7 Ludwig Colon Cancer Initiative Laboratory, Ludwig Institute for Cancer Research, Melbourne, Australia; 8 Faculty of Medicine, Dentistry and Health Sciences, Department of Surgery, University of Melbourne, Melbourne, Australia; 9 Department of Medical Oncology, Royal Melbourne and Western Hospital, Melbourne, Australia; 10 Department of Oncology, Vejle Hospital, University of Southern Denmark, Vejle, Denmark; 11 Biotech Research and Innovation Centre (BRIC), University of Copenhagen, Copenhagen, Denmark; 12 Department of Pathology and Genetics, Pomeranian Medical University, Szczecin, Poland; 13 Department of Pathology, Aarhus University Hospital, University of Aarhus, Aarhus, Denmark; 14 Department of Surgery, Aarhus University Hospital, University of Aarhus, Aarhus, Denmark; The University of Hong Kong, China

## Abstract

MicroRNAs (miRNAs) play a critical role in many biological processes and are aberrantly expressed in human cancers. Particular miRNAs function either as tumor suppressors or oncogenes and appear to have diagnostic and prognostic significance. Although numerous miRNAs are dys-regulated in colorectal cancer (CRC) only a small fraction has been characterized functionally. Using high-throughput functional screening and miRNA profiling of clinical samples the present study aims at identifying miRNAs important for the control of cellular growth and/or apoptosis in CRC. The high-throughput functional screening was carried out in six CRC cell lines transfected with a pre-miR library including 319 synthetic human pre-miRs. Phenotypic alterations were evaluated by immunostaining of cleaved cPARP (apoptosis) or MKI67 (proliferation). Additionally, TaqMan Human MicroRNA Array Set v2.0 was used to profile the expression of 667 miRNAs in 14 normal colon mucosa and 46 microsatellite stable stage II CRC patients. Among the miRNAs that induced growth arrest and apoptosis in the CRC cell lines, and at same time were dys-regulated in the clinical samples, miR-375 was selected for further analysis. Independent *in vitro* analysis of transient and stable transfected CRC cell lines confirmed that miR-375 reduces cell viability through the induction of apoptotic death. We identified YAP1 as a direct miR-375 target in CRC and show that HELLS and NOLC1 are down-stream targets. Knock-down of YAP1 mimicked the phenotype induced by miR-375 over-expression indicating that miR-375 most likely exerts its pro-apoptotic role through YAP1 and its anti-apoptotic down-stream targets BIRC5 and BCL2L1. Finally, *in vivo* analysis of mouse xenograft tumors showed that miR-375 expression significantly reduced tumor growth. We conclude that the high-throughput screening successfully identified miRNAs that induce apoptosis and/or inhibit proliferation in CRC cells. Finally, combining the functional screening with profiling of CRC tissue samples we identified clinically relevant miRNAs and miRNA targets in CRC.

## Introduction

Colorectal cancer (CRC) is a common malignant disease and a leading cause of cancer mortality worldwide. The lifetime risk is about 5% and rising [1]. CRC is caused by the accumulation of numerous genetic and epigenetic alterations. Chromosomal instability leading to allelic imbalance accounts for 70–85% of the tumors whereas 20-15% have DNA mismatch repair defects leading to microsatellite instability. The molecular alterations in CRC have been intensively studied in order to discover diagnostic and prognostic markers. Among others, mRNA expression profiling has been widely used to identify differentially expressed genes with prognostic and diagnostic implications. However, at present none of these have been translated into clinical practice and consequently, there is still a need for further molecular characterization and classification of CRC.

MicroRNAs (miRNAs) comprise an abundant class of small (19–24 nt), non-coding regulatory RNA molecules [2]. They play a critical role in the control of gene expression at the post-transcriptional level by complementary binding of the miRNA strand to the mRNA target sequence, leading to either mRNA degradation or translational inhibition [3]. More than 60% of all protein coding genes contain conserved miRNA binding sites and are thus potential targets of miRNAs [4]. MiRNAs have been shown to be involved in many biological processes such as cell proliferation, apoptosis, differentiation and angiogenesis [5]–[7]. At present, miRNAs have been shown to play important roles in many types of cancers (reviewed by Garzon et al. [8]). The role of miRNAs in the development of CRC has been intensively studied. In 2006, Cummins and co-workers published the first detailed and systematic analysis of miRNA expression in CRC, showing up and down regulation of specific miRNAs [9]. Since then several studies have confirmed the dys-regulation of miRNAs in CRC [10]–[15]. Nevertheless, our knowledge about the function of the individual miRNAs is limited to a fairly small number of miRNAs. Functional screening has been used to identify miRNAs that are causally linked to specific phenotypes (reviewed by Izumiya et al. [16]). In CRC, functional screening has been used in a few cases to identify miRNAs affecting cell proliferation and death [17], [18]. However, the study performed by Nakano et al. was carried out in one cell line only and their findings were not correlated to the expression of the miRNAs in clinical CRC samples. Finally, functional screening has identified clinically relevant miRNAs in pancreatic and testicular germ cell tumors [19], [20].

The acknowledged diagnostic and prognostic potential of miRNAs and a need for further systematic functional analyses of miRNAs in CRC encouraged us to combine high-throughput functional screening with miRNA expression profiling in clinical samples. Ectopic expression of 319 miRNAs in 6 different CRC cell lines was combined with miRNA expression profiling of 14 normal colon mucosa and 46 colorectal tumors. These analyses identified a number of miRNAs that were shown to be differentially expressed in CRC and to have an impact on cellular proliferation and/or apoptosis *in vitro*. Among them miR-375 was selected for further *in vitro* and *in vivo* analyses, which confirmed that miR-375 reduces tumor growth through the induction of apoptotic death. To identify potential miR-375 mRNA targets the expression of miR-375 was correlated to genome-wide mRNA expression profiles. Correlation analysis of both *in vitro* model systems and clinical CRC samples revealed that expression of Yes-associated protein 1 (YAP1) was negatively correlated to miR-375. Ago2 immunoprecipitation indicated that YAP1 is a direct miR-375 target in CRC. Further functional studies indicated that the pro-apoptotic role of miR-375 most likely is mediated by YAP1 and its anti-apoptotic down-stream targets BIRC5 and BCL2L. Finally, lymphoid-specific helicase (HELLS) and nucleolar and coiled-body phosphor protein 1 (NOLC1) were identified as down-stream targets of miR-375 potentially playing a role in cell cycle regulating pathways.

## Materials and Methods

A complete description of the materials and methods is provided in the Supplementary Material (Methods S1).

### Ethics Statement

The use of the human tissue samples for research purpose was approved by the Research Board at the Walter and Eliza Hall Institute of Medical Research HREC (Cohort 1 AUS; HREC No 12/19), the Ethics Committee of the University of Pomeranian (Cohort 1 POL; BN-001/174/05), the Central Denmark Region Committees on Biomedical Research Ethics (Cohort 1 DK; 1999/4678) and the Southern Denmark Region Committees on Biomedical Research Ethics (Cohort 2 DK; VF-20040047). Informed written consent was given by all participants. All animal experiments were approved by the Danish Animal Experiments Inspectorate (file number: 2013-15-2934-00861/ACHOV)

### Clinical samples and cell lines

Cohort 1 consisted of a total of 46 fresh frozen microsatellite stable (MSS), primary stage II (T2-4, N0, M0) CRCs and 14 normal colon mucosa. An independent cohort consisting of 25 normal colon mucosa and 63 MSS, primary stage I–IV (T2-4, N0-3, M0/1) CRCs (cohort 2) was used for validation. Patients and sample characteristics are summarized in Table 1. Cell lines, growth conditions and cell line authentication can be found in the Supplementary Material (Methods S1).

**Table 1:** Summary of patients and sample characteristics.

	Cohort 1 (n = 60)^1^		Cohort 2 (n = 88)^2^	
	Normal mucosa	Adenocarcinoma	Normal mucosa	Adenocarcinoma
**Nationality**				
Denmark	14	29	25	63
Poland	-	8	-	-
Australia	-	9	-	-
**Gender**				
Female	6	18	13	35
Male	8	28	12	28
**Microsatellite status**				
MSS	-	46	-	63
**Stage (TNM)**				
I	-	-	-	3
II	-	46	-	24
III	-	-	-	19
IV	-	-		17
**Age of onset (median, range/years)**	-	67 (53–86)	-	71 (47–90)
**Tumor location**				
Colon	-	22	-	43
Rectum	-	24	-	20
**Histological type**				
Adenocarcinoma	-	44	-	56
Mucinous	-	2	-	5
Other	-	-		2
**Differentiation (dominating grade)**				
High	-	7	-	1
Moderate	-	36	-	49
Low	-	3	-	12
Unknown	-	-	-	1
**Cancer cells in sample (median, range/%)**	-	80 (50–90)	-	-3
**RNA quality (Median, range/RIN score)**	9.0 (7.6–10.0)	9.1 (5.1–10.0)	-4	-

1 Samples were collected from 1999–2006.

2 Samples were collected from 2004–2005.

3 The tumor samples were collected in RNAlater and hence the amount of cancer cells could not be predicted.

4 RIN scores are not available for the cohort 2.

### Animals

BALB/cAnNTac-nude immune deficient mice (6–8 weeks, 20–22 g) were purchased from Taconic Europe (DK-4623 Ll. Skensved, Denmark). Prior to the experiments, the mice were afforded an adaptation period of at least 14 days. The mice were maintained at identical conditions (i.e. constant room temperature (22°C) and a natural day/night light cycle. Standard laboratory food and water were provided ad libitum.

### MiRNA functional library screen

The cell spot microarray technology was used to generate high density pre-miRNA transfection microarrays as previously described with minor modifications [21]. Briefly, a library holding 319 synthetic human pre-miRNAs (Ambion pre-miR v1.0) (Ambion, Austin, TX, USA) was used for printing of the arrays. Subsequently, the CRC cells (HCT116, LS174T TR4, DLD1 TR7, HT29, Caco2 and SW480) were seeded onto the arrays and reverse transfected for 48 hours. To allow microscopic detection of pre-miRNAs effects influencing cell proliferation and/or apoptosis the arrays were immunostained for Ki-67 (proliferation marker) and for cleaved poly ADP-ribose polymerase (cPARP) (apoptosis marker). DNA was stained using 4′,6 Diamidino-2-phenylindole (DAPI)(Invitrogen, Carlsbad, CA, USA) or SYTO60 (Invitrogen). The microarray analysis was performed with microscopic imaging of the arrays using scanR high content imager (Olympus) and the effect of miRNA over-expression on apoptosis and cell proliferation was considered as previously described [21]. Briefly, after normalization a z-score (z = (χ-μ)/σ) was calculated for scoring of the measured spot values. χ = normalized spot level value, μ = global array mean and σ = standard deviation (sd).

### RNA and miRNA isolation

Total RNA (>200 bases) was isolated from cell pellets and fresh frozen tissue sample using RNeasy Mini Kit (Qiagen), according to the manufacturer's instructions. The small RNAs (<200 bases) were recovered from the flow-through fraction using RNeasy Micro Kit together with the RNeasy MinElute spin columns (Qiagen)(described in the Supplementary Material (Methods S1)). The small RNAs from the RNAlater preserved tissue samples were isolated directly using the RNeasy Micro Kit (Qiagen).

### miRNA profiling in clinical samples

The miRNA expression profiling was performed using the stem loop RT-qPCR based TaqMan Human MicroRNA Array Set v2.0 as indicated by the manufacturer (Applied Biosystems, Carlsbad, CA, USA) [22]. The NormFinder algorithm was used to identify appropriate reference genes [23].

### miRNA and mRNA RT-qPCR

Single tube TaqMan microRNA or mRNA assays (Applied Biosystems) were used to quantify individual mature miRNAs or mRNAs (details in Supplementary Material (Methods S1)). The Applied Biosystems TaqMan Assay ID's and the primer used for detection of the mRNA reference gene ubiquitin C (UBC) are listed in Table S1 in File S1.

### MTT assay

Cells were seeded in 96-well plates, reverse-transfected and incubated for 72 hours with pre-miRs or siRNAs. Cell viability/proliferation was measured using 3-[4,5-dimethylthiazol-2-yl]-2.5-diphenyltetrazolium bromide (MTT) assay (Roche Applied Science, Penzberg, Germany)(described in the Supplementary Material (Methods S1)). The Applied Biosystems pre-miR and *Silencer* Select siRNA ID's and the sequences of the YAP1 siRNAs from GenePharma (Shanghai, China) are listed in Table S2 in File S1.

### LDH assay

Cells were seeded in 96-well plates and reverse-transfected for 48 or 72 hours with pre-miRs or siRNAs (Table S2 in File S1) (further details are found in the Supplementary Material (Methods S1)). Subsequently, cellular death (LDH activity) was measured using the Cytotoxicity Detection Kit^PLUS^(LDH)(Roche Applied Science).

### Caspase 3/7 activity assay

The Caspase 3/7 activity assay was used to measure apoptotic death and performed mainly as described previously [24]. Briefly, the cells were seeded in 24 well plates and reverse-transfected with pre-miRs or siRNAs for 48 hours (Table S2 in File S1). The Caspase 3/7 inhibitor (z-DEVD-fmk) (Biovision, San Francisco, CA, USA) was added 6 hours post-transfection (final concentration 25 µM). Caspase 3/7 activity in cell lysates, measured by the liberation of AFC (excitation, 400 nm; emission 489 nm) from the substrate Ac-DEVD-AFC (Biomol, Plymouth Meeting, PA, USA), was measured using a multiplate reader; Multiscan MCC/340 (ThermoFisher Scientific).

### Laser Capture Microdissection

Laser Capture Microdissection (LCM) was performed on cryosections from paired cancer and adjacent normal colon mucosa biopsies (described in detail in the Supplementary Material (Methods S1)). The epithelial cells were captured on individual caps with the Veritas 704 Microdissection Instrument (Applied Biosystems) using ultraviolet laser cutting according to the instructions given by the manufacturer.

### The MIR-375 methylation levels in CRC cell lines and clinical samples

Bisulfite modified DNA was whole genome amplified and hybridized to Infinium HumanMethylation450 BeadChips (Illumina, San Diego, CA) overnight as described by the manufacturer. BeadChips were scanned with a BeadXpress Reader instrument (Illumina) and data analyzed using Bead Studio Methylation Module Software (Illumina). Methylation levels were provided in beta values, with a beta value of 0 corresponding to no methylation, and 1 corresponding to full methylation. The IDs of the CpG sites in close proximity to *MIR-375* were as follows CpG1; cg00215432, CpG2; cg00218620, CpG3; cg00705280, CpG4; cg02257674, CpG5; cg04348419, CpG6; cg06214770, CpG7; cg14358282, CpG8; cg21615583, CpG9; cg22306928, CpG10; cg01822124 and CpG11; cg26394220.

### ChIP analysis

The ChIP analysis was performed as described previously [25]. The primers used to amplify the ChIP DNA regions are listed in Table S1 in File S1.

### Identification of potential miR-375 targets based on mRNA profiling and *in silico* target prediction

The mRNA profiling of miR-375 transfected HCT116 cells and the clinical samples are described in the Supplementary Material (Methods S1). The location and number of miR-375 seed sequences (i.e. complementary to the position 2–8 of the miRNA) within the full length mRNA sequence were mapped using sequence data retrieved from TargetScan v5.2 and Ensembl 62 databases [26], [27].

### Ago2 immunoprecipitation

Scr and miR-375 transfected cell (∼3.5×10^6^) were scraped of culture flasks on ice in gentle lysis buffer (20 mM TRIS pH 7.5, 10 mM NaCl, 0.5% NP-40, 2 mM EDTA supplemented with RNase inhibitor RNaseOut (Invitrogen) and Complete Mini Protease Inhibitor Cocktail (Roche)) and hypertonically lysed by increasing the NaCl concentration to 150 mM. After centrifugation at 4°C and 19,000*g for 10 minutes the supernatant was collected and subjected to immunoprecipitation (10% was used for input control) by incubation with monoclonal Ago2 antibody (11A9) (Sigma-Aldrich) -bound Protein G-coupled Magnetic Dynabeads (Life technologies) (15 mg 11A9 per 25 ml beads) following the manufacturer's recommendation. Anti-FLAG immunoprecipitation was done in parallel as a negative control (antibody F1804, Sigma). The beads were washed 5 times in ice cold washing buffer (50 mM TRIS pH 7.5, 150 mM NaCl and 0.05% NP-40). Total RNA from input, Ago2-IP and FLAG-IP complexes was purified using QIAZol (Qiagen).

### Ingenuity Pathway Analysis

Ingenuity Pathway Analysis (IPA) software (Ingenuity Systems, Redwood city, CA, USA) was used to gain insight into the overall biological changes introduced by the ectopic expression of miR-375. Normalized and filtered mRNA data were uploaded to IPA. Using the Ingenuity Pathways Knowledge Base (IPKB) each gene was linked to specific functions, pathways and diseases and an enrichment analysis was performed examining whether the data were enriched for genes associated with a particular function. Fisher's exact test was applied to evaluate the significance of the enrichments

### Protein extraction and Western blotting

Protein extraction and Western blotting analysis were performed according to standard procedures (details in Supplementary Material (Methods S1)).

### Construction of plasmids for the Luciferase reporter assay

Selected fragments of the 3′UTRs of HELLS (NM_018063) and NOLC1 (NM_004741) containing putative miR-375 binding sites were amplified from normal human genomic DNA and cloned downstream of the *Renilla* Luciferase gene in the siCHECK-2 vector (Promega, Fitchburg, WI, USA). Selected constructs were mutated in the putative miRNA binding region using QuickChange Lightning Site Directed Mutagenesis Kit (Agilent Technologies) according to the provided protocol. Details are provided in the Supplementary Material (Methods S1) and the primers used for cloning and site directed mutagenesis are described in Table S3 in File S1.

### Luciferase reporter assay

Transfected HEK-293T cells were analyzed using Dual Glo Luciferase Assay System (Promega) as described previously [28] (details are described in the Supplementary Material (Methods S1)). Luminescence was detected with a multiplate reader; Multiscan MCC/340 (ThermoFisher Scientific, Waltham, MA, USA). The *Renilla* Luciferase activity was normalized to the *Firefly* Luciferase activity for each transfected well, to correct for differences in transfection and harvest efficiencies.

### Generation and characterization of stable HCT116 cells with inducible miR-375 expression

The generation of stable HCT116 cells with inducible expression of miR-375 is described in detail in the Supplementary Material (Methods S1). Initially, *MIR-375* was cloned into the 3′ UTR region of the *turbo red fluorescence protein gene* (tRFP) of the pSBInducer10 vector (*MIR375*_pSBInducer10). The pSBInducer10 vector was constructed by replacing lentiviral elements in the pINDUCER vector [29] with SleepingBeauty inverted terminal repeats. Stable HCT116_miR-375 and HCT116_Scr cell pools were generated as follows. Firstly, 1.5×10^6^ HCT116 cells were transfected with pCMV-SB100XCO (vector with transposase) and either *MIR375*_pSBInducer10 (HCT116_miR-375) or *Scr*_pSBInducer10 (HCT116_scr). Forty-eight hours post-transfection puromycin (final concentration 1 µg/mL) (Sigma) was added to select for stably transfected cells. The puromycin selection was carried out for 5 days. Subsequently, the cells were treated with 50 ug/ml doxycycline (dox) (Sigma) for 48 hours leading to transcriptional activation of the tRFP-*MIR375* cassette. The tRFP fluorescence marker was used as a surrogate to sort for cell populations expressing the highest level miR-375 after induction of dox. Briefly, the cells with the highest tRFP level (100–1000 times above the background level in untreated cells) (HCT116_miR-375H and HCT116_ScrH) were isolated by fluorescence-activated cell sorting (FACS) using a 4-laser FACSAriaIII (BD Biosciences, San Jose, CA) and used for all subsequent analyses. Dox dependent expression of mature miR-375 in the HCT116_miR-375H cells was analyzed using RT-qPCR as describe earlier. The HCT116_miR-375H cells were phenotypically characterized using xCELLigence (Roche Applied Science), Caspase 3/7 assays and YAP1 Western blotting (details in Supplementary Material (Methods S1)).

### *In vivo* tumor growth analysis

HCT116_miR-375H cells (3.5×10^6^ cells per injection) were suspended in 200 µl PBS and injected subcutaneously into the left flank of anesthetized mice (n = 12). The mice were divided into two groups with 6 animals in each: Group A; miR-375 (+ dox) and Group B; control (− dox). The tumors were measured regularly (length and width) by the same observer. To induce expression of miR-375, dox (Vibradox Sandoz)(0,2 mg/ml) was added to the drinking water of the mice in group A, when the tumors reached a size of approximately 50 mm^3^. The mice in group B were given ordinary drinking water. The dox treatment was carried out for 14 days. Four mice were taken out of the study prior to the dox treatment (two had very fast growing tumors and two had tumors that stopped growing) i.e. n = 4 in Group A and B. The estimated tumor volume (EV) was calculated as EV = length×(width)^2^×1/2. EV was plotted against the number of days after initiation of dox treatment and xenograft growth curves were generated.

### Statistical analysis

The significance of miRNA expression changes were analyzed using the Mann Whitney U test in the Multi Experiment Viewer (MeV) array analyzer software [30]. The hierarchical clustering of differentially expressed miRNAs was performed using Gene Cluster 3.0 [31]. Only, miRNAs that were expressed in more than 80% of the samples were included. Heat maps were generated with Java TreeView [32]. Student's unpaired *t*-test was applied to compare miRNA induced changes with respective controls in the MTT assay, LDH assay, Apoptosis assay, RT-qPCR analysis and *in vivo* tumor growth analysis. The Student's paired t-test was used for the RT-qPCR analysis of microdissected tissue samples. A *p*-value<0.05 was considered statistically significant.

## Results

### High-throughput screening identifies a series of miRNAs that induces apoptosis and/or inhibits proliferation in CRC cell lines

To identify miRNAs with the most consistent impact on growth and/or survival of CRC cancer cells we performed a high-throughput screening in six CRC cell lines, using a library holding 319 synthetic human pre-miRNAs. Pre-miR induced changes in cPARP and Ki-67 were used to identify miRNAs that induced apoptosis and/or inhibit proliferation respectively. Distributions of the z-scores for each pre-miR are shown in Figure S1. The z-score rank products were used to identify consistency of the pre-miRNA effects across all tested CRC cell lines [33]. The rank product Top-40 pre-miRs are listed in Table S4 in File S2 (increased cPARP) and Table S5 in File S2 (reduced Ki-67). Several of the Top-40 ranked miRNAs have previously been shown to affect either apoptosis and/or proliferation *in vitro* (a subset of these are shown in Table S6 in File S1).

### miRNA expression profiling of clinical samples

**Figure 1:**
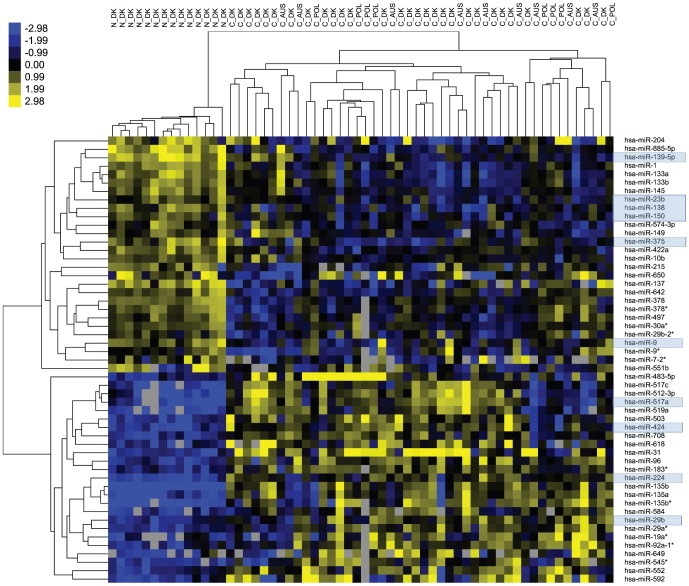
miRNAs differentially expressed in normal colon mucosa versus stage II CRC. Hierarchical clustering of miRNAs with a significant different expression between colorectal adenocarcinomas and normal colon mucosa samples (p≤0.01). The rows represent individual miRNAs and the columns represent individual tissue samples. The scale represents the intensity of the gene expression (log2 scale ranging between −2.98 and 2.98) (p-values and FCs are found in Table 2). The miRNAs marked with blue were found to inhibit proliferation and/or induce apoptosis in the high-throughput screen (Top-40 ranked miRNAs).

To address the *in vivo s*ignificance of the miRNAs identified in the high-throughput functional screening we profiled the expression of 667 unique miRNAs in 14 normal colon mucosa and tissue samples from 46 clinical stage II CRC samples. In total, 53 miRNAs were differentially expressed between normal mucosa and CRC (Mann Whitney U test p≤0.01 and 1.5≤FC_(log2)_≤−1.5) (Figure 1 and Table 2). A subset of 25 miRNAs showed increased expression in CRC tumors relative to normal mucosa whereas 28 miRNA were down-regulated in the CRC tumors. Combining the results of the miRNA profiling of clinical samples with the results of the high-throughput functional screening we identified 10 miRNAs that were differentially expressed in clinical CRC samples and at the same time induced phenotypic changes in the functional screening (Top-40 ranked) (Table 2 and Table S6 in File S1). Additionally, eight dys-regulated miRNAs induced phenotypic changes in at least one cell line, but were not Top-40 ranked (Table 2). Eleven of the above miRNAs were down-regulated and hence these miRNAs are tumor suppressor candidates. Finally, 20 of the dys-regulated miRNAs were not included in the pre-miRNA library from Ambion and hence their ability to induce phenotypic changes cannot be analyzed in the present study (Table 2). In conclusion, we identified 11 miRNAs that were down-regulated in CRC samples and at the same time induced proliferation and/or inhibited apoptosis in CRC cell lines and hence these miRNAs are potentially involved in colorectal tumorigenesis.

**Table 2:** The most differentially expressed miRNAs in stage II colorectal adenocarcinomas and their ability to induce phenotypic changes in the high-throughput analysis.

A-array				B-array			
miRNA id	Phenotype	p-value	FC_(log2)_ 1	miRNA id	Phenotype	p-value	FC_(log2)_ 1
**Up-regulated**				**Up-regulated**			
hsa-miR-135b	-	5.46E-08	4.60	hsa-miR-135b*	NA	1.53E-07	4.27
hsa-miR-517c	(+)P	1.57E-05	3.45	hsa-miR-649	NA	5.12E-05	2.38
hsa-miR-512-3p	-	4.34E-05	3.02	hsa-miR-592	NA	0.0009	1.90
hsa-miR-31	-	2.17E-05	2.96	hsa-miR-545*	NA	2.63E-05	1.86
hsa-miR-519a	-	9.71E-06	2.83	hsa-miR-584	NA	0.0005	1.74
hsa-miR-135a	(+)A	9.05E-07	2.81	hsa-miR-29a*	NA	6.02E-06	1.69
hsa-miR-483-5p	-	0.001	2.79	hsa-miR-92a-1*	NA	5.00E-05	1.60
hsa-miR-517a	+A	1.21E-05	2.74	hsa-miR-552	NA	6.58E-05	1.59
hsa-miR-503	(+)P	1.68E-06	2.11	hsa-miR-19a*	NA	0.008	1.55
hsa-miR-224	+A	2.30E-07	2.10	hsa-miR-183*	NA	6.55E-06	1.52
hsa-miR-96	-	2.00E-06	2.05				
hsa-miR-424	+P	6.92E-07	2.03	**Down-regulated**			
hsa-miR-618	NA	0.0001	1.81	hsa-miR-378	-	1.99E-08	−2.20
hsa-miR-708	NA	8.32E-06	1.67	hsa-miR-378*	NA	2.16E-07	−2.07
hsa-miR-29b	+P	4.67E-06	1.52	hsa-miR-650	NA	0.002	−2.03
				hsa-miR-497	-	1.49E-05	−1.71
**Down-regulated**				hsa-miR-30a*	(+)P	1.96E-07	−1.70
hsa-miR-885-5p	-	8.07E-08	−3.09	hsa-miR-7-2*	NA	0.002	−1.60
hsa-miR-137	-	4.38E-07	−2.93	hsa-miR-9*	-	0.0002	−1.58
hsa-miR-139-5p	+A	8.07E-08	−2.92	hsa-miR-29b-2*	NA	0.0001	−1.53
hsa-miR-133a	-	1.43E-07	−2.66				
hsa-miR-1	-	8.89E-08	−2.59				
hsa-miR-133b	-	2.30E-07	−2.39				
hsa-miR-375	+A	6.32E-07	−2.38				
hsa-miR-138	+P	2.30E-07	−2.32				
hsa-miR-204	-	0.0009	−2.24				
hsa-miR-145	(+)P	2.093E-07	−2.19				
hsa-miR-422a	(+)P	6.02E-08	−2.16				
hsa-miR-642	Na	6.64E-08	−2.09				
hsa-miR-9	+P	1.18E-06	−2.03				
hsa-miR-215	-	1.10E-05	−2.03				
hsa-miR-150	+A	2.58E-06	−1.75				
hsa-miR-23b	+P	2.37E-06	−1.69				
hsa-miR-10b	(+)A	4.81E-07	−1.68				
hsa-miR-574-3p	NA	2.73E-05	−1.63				
hsa-miR-149	(+)A	0.0001	−1.53				
hsa-miR-551b	NA	0.006	−1.53				

1 A FC _(log2)_≤−1.50 and ≥1.50 and a p-value≤0.01 was considered significant (Mann-Whitney U test).

NA: miRNAs not included in the pre-miRNA library from Ambion.

+: miRNAs that induced phenotypic changes (Top-40 ranked).

(+): miRNAs that induced phenotypic changes in at least one cell line (not Top-40 ranked).

A: Induction of apoptosis.

P: Inhibition of proliferation.

### Validation of the phenotypes identified in the high-throughput screen

**Figure 2:**
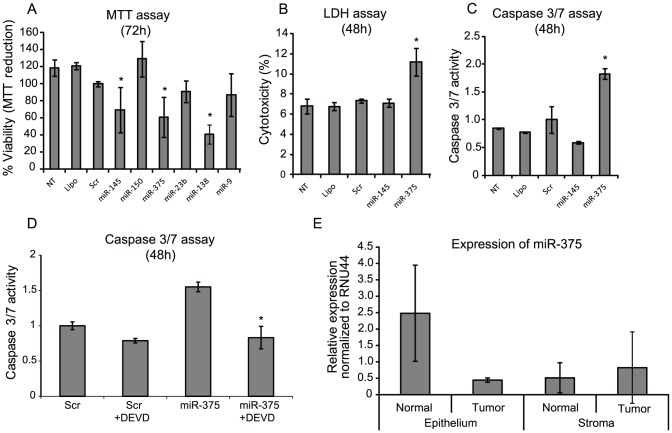
Phenotypic analyses of selected miRNAs in HCT116 cells upon ectopic expression of the miRNAs. (A) Cellular viability (MTT assay): Data are presented as ±sd. of at least 3 independent experiments each with three biological replicates and normalized to Scr. *p-value<0.05 and MTT reduction >20%. (B) Cellular death (LDH release assay): The cellular death was expressed as percentage of released LDH out of total cellular LDH. At least two independent experiments were carried out and performed in triplicates. The result of one representative experiments ±sd. is shown. *p-value<0.05. (C) Induction of apoptosis (Caspase 3/7 activity): The Caspase 3/7 activity in the lysate of pre-miRNA transfected cells was examined by fluorometric kinetic analysis and expressed relative to the Caspase 3/7 activity in “Scr” transfected cells. Data are presented as ±sd. of at least 2 independent experiments each with three biological replicates. *p-value<0.05. (D) Inhibition of miR-375 induced apoptosis by the Caspase 3/7 inhibitor z-DEVD-fmk. The Caspase 3/7 activity was measured as described in (C). Z-DEVD-fmk (DEVD) (25 µM) was added to the cells six hours post-transfection. Non treated cells (NT), Lipofectamine only (Lipo) and pre-miR miRNA Precursor Molecules-Negative Control #1 (Scr) were included as negative controls in all assays. The pre-miR-145 transfected cells were included as a positive control for performance of the MTT assay. (E) The down-regulation of miR-375 is a result of reduced expression in the epithelial cells of the tumor. Expression of miR-375 in laser capture microdissected colorectal cancer tissue. The expression was analyzed in epithelial and stromal cells from paired colorectal adenocarcinomas (n = 3) and adjacent normal (n = 3) colon mucosa LCM biopsies using RT-qPCR. The columns represent the mean expression in three samples ± sd.

To validate the identified phenotypes, the miRNAs that were down-regulated in clinical samples and Top-40 ranked in the phenotype screen (miR-150, miR-375, miR23b, miR-138, miR-139-5p and miR-9) were subjected to detailed functional analysis using HCT116, HT29, LS174T TR4, DLD1 TR7 and SW480 colon cancer cell lines. We have recently published a detailed functional analysis of miR-139-5p and hence miR-139-5p was not included in these analyses [25]. The ectopic expression of miR-375, miR-9 and miR-138 significantly reduced the viability of more than one cell line (MTT reduction >20% and p≤0.05) (Figure 2A (HCT116) and Figure S2), possible due to a general anti-proliferative or pro-apoptotic role of these miRNAs. miR-150 and miR-23b only reduced the viability of one cell line (DLD1 TR7). Among the remaining miRNAs, miR-375 was identified as an apoptosis inducing miRNAs in the high-throughput screen. To validate this finding, we carried out LDH and Caspase 3/7 assays in transfected CRC cell lines. MiR-375 increased Caspase 3/7 activity and demonstrated coincident increase in cellular death (LDH release) in both HCT116 and DLD1 TR7 (Figures 2B and C, and Figure S3). Furthermore, the apoptotic death induced by miR-375 could be inhibited with z-DEVD-fmk (Caspase 3/7 inhibitor) (Figure 2D) demonstrating that the induced apoptosis is dependent on Caspase 3/7 activity. In conclusion, the validation analysis confirmed the anti-proliferative role of miR-9 and miR-138, and the apoptosis inducing capacity of miR-375 as identified in the high-throughput analysis. The above miRNAs have previously been shown to be dys-regulated in human cancers and to reduce proliferation and/or induce apoptosis *in vitro* (Table S6 in File S1). miR-375 and miR-138 have not been functionally characterized in detail in CRC. Finally, miR-375, miR-138 and miR-9 were selected for further analysis due to their down-regulation in clinical samples and their ability to induced phenotypic changes *in vitro*.

### Detection of miR-375, miR-138 and miR-9 in laser microdissected colorectal tissue

To elucidate the cellular origin of miR-375, miR-138 and miR-9, we measured their expression in laser captured microdissected colorectal adenocarcinomas and adjacent normal colon mucosa (Figure 2E and Figure S4). These analyses showed that in normal colon mucosa miR-375 was expressed at a higher level in the epithelial cells than in stromal cells (p = 0.02) (Figure 2E). Whereas in the adenocarcinoma miR-375 was expressed at comparable levels in epithelial and stromal cells (p = 0.27). Additionally, miR-375 expression in normal epithelial cells was significantly higher than the expression in epithelial cells from adenocarcinoma (p = 0.03). Overall, these results indicate that the down-regulation of miR-375 in CRC is a result of a reduction in the miR-375 expression in epithelial cells of the tumor. miR-9 and miR-138 were expressed primarily by stromal cells from both normal colon mucosa and adenocarcinomas (Figure S4). These results are consistent with previously published miRNA expression profiles of laser-microdissected normal mucosa, adenomas and adenocarcinomas [34]. The clearly epithelial origin of the high miR-375 expression in normal colon mucosa and the down-regulation of miR-375 in epithelial cells from adenocarcinomas led to the selection of miR-375 for further analysis.

### Validation of miR-375 down-regulation in clinical samples

The down-regulation of miR-375 in stage II CRC observed in the present study was confirmed in an independent cohort (cohort 2) of 25 normal colon mucosa samples and 63 primary CRCs of different stages (stage I–IV, T2-4, N0-3, M0/1) (Figures S5A and B). miR-375 was significantly down-regulated not only in stage II tumors (p = 0.0002 and absolute FC = 4.9) but also in the cohort as a whole combining tumors of different stages (p = 0.0002 and absolute FC = 4.5). On the contrary, miR-375 was not differentially expressed between the tumors of different stages. Additionally, miR-375 has previously been shown to be down-regulated in different cohorts of CRC samples [35], [36] and in several other human cancers (see Table S6 in File S1 for references) indicating that the down-regulation of miR-375 is a general event in tumor development.

### *MIR-375* methylation analysis in CRC cell lines and clinical CRC samples

**Figure 3:**
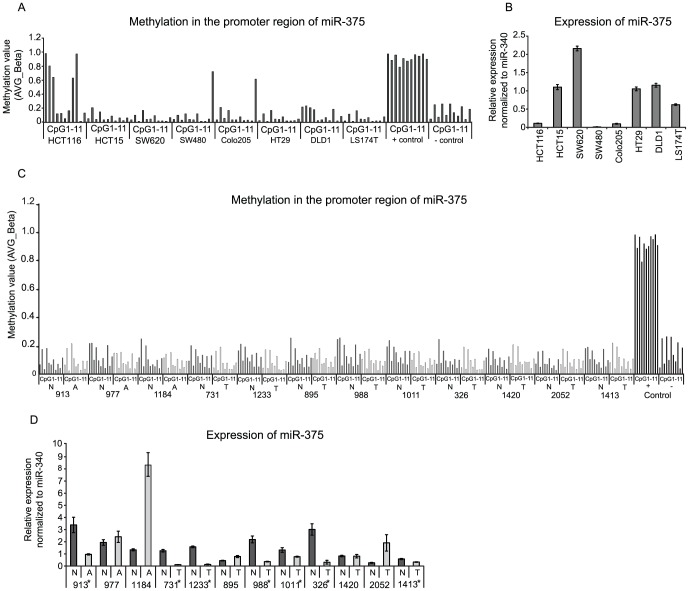
Methylation of *MIR-375 in* CRC cell lines and clinical samples using Infinium HumanMethylation450 BeadChips. (A and C) Methylation of *MIR-375* in 8 CRC cell lines (A) and 12 normal colon mucosa samples paired with colorectal adenomas (n = 3) or adenocarcinomas (n = 9) (C). CpG sites in close proximity to *MIR-375* (CpG1-11) were analyzed. (B and D) Expression analysis of miR-375 in the CRC cells lines (B) and the paired clinical samples (RT-qPCR) (D). The miR-375 expression was measured in triplicates and normalized to miR-340. * indicate the pairs of clinical samples with significant miR-375 down-regulation (FC_(log2)_>1.5). N: normal colon mucosa, A: adenoma and C: adenocarcinoma.

miR-375 is an intergenic miRNA and is associated with a CpG island indicating that this miRNA may be down-regulated by epigenetic silencing. Previous studies have shown that miR-375 is indeed down-regulated due to hypermethylation in esophageal and breast cancer [37]–[40]. Hypermethylation of *MIR-375* has also been demonstrated in melanoma and in the CRC cell line HCT116 cell [39], [40]. These data encouraged us to study the methylation of *MIR-375* in CRC cell lines and clinical CRC tissue samples using Infinium HumanMethylation450 BeadChips. We specifically look at the methylation level of 11 CpG sites situated in close vicinity to the pri-miR-375 transcription start site [41]. These CpG sites are situated mainly in the genomic regions that have previously been analyzed using bisulfite sequencing [38], [40], [42]. Methylation analysis of eight CRC cell lines confirmed a high methylation level of 5/11 CpG sites in HCT116. Additionally, SW480 and Colo205 were highly methylated at 1/11 CpG sites whereas the other cells lines demonstrated no methylation of the 11 CpG sites (Figure 3A). miR-375 expression analysis demonstrated that HCT116, SW480 and Colo205 all exhibited lower expression of miR-375 than the cell lines with no methylation (Figure 3B). On the contrary, Infinium HumanMethylation450 BeadChip methylation analysis of normal colon mucosa with paired adenomas or adenocarcinomas did not identify any hypermethylation of *MIR-375* in the adenomas and adenocarcinomas (Figure 3C), although miR-375 was down-regulated (FC (log2)>1.5) in 7/12 pairs (Figure 3D). These results indicate that epigenetic silencing of *MIR-375* is not the general mechanism of miR-375 down-regulation in CRC.

### Regulation of miR-375 by β-catenin/TCF4 activity

A previous study, has suggested a direct link between β-catenin activation and miR-375 repression in hepatocellular tumors [43]. Furthermore, results from our laboratory have shown that miR-375 is up-regulated upon inhibition of β-catenin/TCF4 activity in the dox inducible dominant negative (dn)TCF4 DLD1 cell line (DLD TR7), which has been used as a model to study Wnt regulation of miRNAs in CRC [25]. We therefore asked whether we could detect chromatin occupancy of β-catenin/TCF4 complexes at TCF4 sites in proximity to the miR-375 hairpin. We identified two TCF4 sites within 80–90 kb of the miR-375 hairpin and analyzed the binding of TCF4 to both sites using a chromatin immunoprecipitation (ChIP) approach with polyclonal TCF4 antibody in DLD1 TR7 cells (Figure S6). MYC 3′ enhancer region located at 3′ of the MYC gene has previously been reported to be bound by TCF4 (positive control) whereas the exon 2 of the myoglobin gene is not bound by TCF4 (negative control) [44], [45]. Although, we did find that TCF4 bound to the MYC enhancer region, we did not detect any TCF4 binding at the *MIR-375* locus. Hence our data do not support the hypothesis that miR-375 expression is directly modulated by chromatin-bound β-catenin/TCF4 complexes.

### Identification of miR-375 targets using transcription profiling

**Figure 4:**
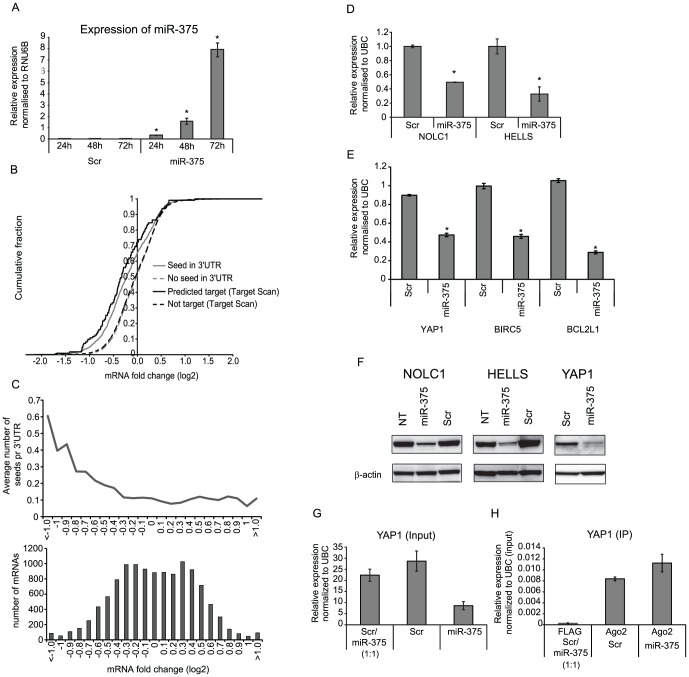
mRNA profiling of HCT116 cells upon ectopic expression of miR-375 and miR-375 target identification. (A) Reconstitution of mature miR-375 upon transfection with pre-miR-375 or Scr (RT-qPCR). (B) Cumulative fraction plotted as a function of log2 fold changes. The mRNAs were dichotomized according to the presence or absence of minimum one seed match in the 3′UTR or according to target prediction using Target Scan v5.2. The mRNAs with minimum one 7mer-m8 seed match within the 3′ UTR showed a higher propensity to down-regulation upon miR-375 over-expression. (C) The mRNAs were ranked according to fold change and grouped into a total of 23 bins. Upper panel: The average 7mer-8m seed frequency within the 3′UTR regions in each bin was calculated. Bottom panel: Overall miRNA-induced mRNA fold change. (D and E) Relative expression of HELLS, NOLC1, YAP1, BIRC5 and BCL2L1 upon ectopic miR-375 expression using RT-qPCR. (F) Western blots demonstrating the effect of miR-375 on the protein level of HELLS, NOLC1 and YAP1 in HCT116 cells. Loading control: β-actin. *p<0.05. (G–H) Ago 2 immunoprecipitation. (G) RT-qPCR expression analysis of YAP1 in the cell lysates of miR-375 or Scr transfected cells (input) used for Ago2 immunoprecipitation. (H) Ago2 immunoprecipitation from cell lysates of miR-375 or Scr transfected cells (IP) followed by YAP1 expression analysis using RT-qPCR. Immunoprecipitation with a FLAG antibody was used as negative control. A 1∶1 ratio of the lysates from miR-375 and Scr transfected cells was used for FLAG immunoprecipitation. The columns represent the mean of 3 replicates ± sd.

In order to elucidate the mechanism behind the induction of apoptotic death by miR-375 we set out to identify miR-375 targets. Initially, we analyzed the expression of mature miR-375 in HCT116 transfected with a miR-375 mimic at different time points post transfection (Figure 4A). A significant up-regulation of mature miR-375 was observed 24 hours post-transfection. Subsequently, genome wide microarray transcription profiling of HCT116 cells over-expressing miR-375 was carried out to screen for target candidates regulated at the transcriptional level. As expected, more mRNAs were down-regulated than up-regulated as a result of miR-375 ectopic expression (Figure 4B). Integration of the transcription data with *in silico* target prediction revealed, that miRNAs harboring miR-375 seed matches in the 3′UTR were more frequently down-regulated than mRNAs with no seed match (Figure 4B). Furthermore, the average number of 7mer-m8 motifs per 3′UTR was enriched among down-regulated mRNAs (Figure 4C). The same tendency was also evident when the target region was expanded to the full length mRNA (data not shown), which is in agreement with functional target sites also being located within open reading frames [46]. To gain insight into the over-all biological changes introduced by the ectopic expression of miR-375, the most effected transcripts (p-value<0.05, FC_(log2)_<−1.0 or >1.0 (206 genes) or FC_(log2)_<−0.5 or >0.5 (1236 genes)) were analyzed using the Ingenuity Pathway analysis (IPA) software. This analysis demonstrated significant associations to specific biological functions such as cell death, cell cycle, cellular growth and, proliferation, which are all highly relevant for the observed growth inhibitory phenotype (Figure S7). Subsequently, we analyzed whether mRNAs that had previously been identified as direct miR-375 targets in other tissue were affected in HCT116 cells upon up-regulation of miR-375. Using TarBase6.0, we identified nine mRNAs, which had all been identified as direct miR-375 targets using Luciferase reporter assay (Table 3) [47]. Interestingly, six of these known miR-375 targets were also significantly down-regulated in HCT116 upon miR-375 ectopic expression. The remaining three targets were either not expressed in HCT116 (median log intensity <7) (1/8) or not present on the array (2/8) (Table 3). The above results support the notion that identical miRNA targets respond in similar ways to miRNA overexpression in cell lines originating from various tissues.

**Table 3:** Expression of known direct miR-375 targets.

Gene symbol	FC_HCT116_miR-375_ (log2)	p-value (corrected)	FC_clinical CRC samples_ (log2)Δ	p-value (corrected)	Pearson	Tissue	Reference
JAK2	−1.03	0.05	−0.4	0.05	0.6	Gastric^(Hs)^ * Neuroblastoma^(Mm)^ ¤	[50], [55]
HuD (ELAVL4)	ND	-	-	-	-	Neurites	[61]
YWHAZ (14-3-3ζ)	−1.03	0.02	0.5	NS	−0.1∧	Gastric^(Hs)^	[52]
*USP1*	*−0.7*	*0.01*	*0.6*	*0.02*	*−0.4* ∧	*Gastric* ^(Hs)^ *Neuroblastoma* ^(Mm)^	[52], [55]
C1QBP‡	-	-	-	-	-	Gastric^(Hs)^, Neuroblastoma^(Mm)^	[52], [55]
MTPN‡	-	-	-	-	-	Pancreas^(Hs)^ Neuroplastoma^(Mm)^	[55], [62]
*YAP1*	*−0.57*	*0.04*	*1.6*	*1.3E-06*	*−0.6*	*Liver* ^(Hs)^	[49]
ADIPOR2	−0.5	0.02	0.1	NS	0.1	Neuroblastoma^(Mm)^	[55]
PDK1	−1.06	0.03	−0.1	NS	0.3	Esophageal^(Hs)^ Gastric^(Hs)^	[37], [52]

Δ Analyzed in 24 normal mucosa and 30 MSS adenocarcinomas that has previously been profiled using Human Exon 1.0 ST arrays (Thorsen K et al. Alternative Splicing of SLC39A14 in Colorectal Cancer is Regulated by the Wnt Pathway,

Molecular and Cellular Proteomics, 2011).

ND: not detected (median log intensity <7).

NS: not significant (a p-value≤0.05 was considered significant).

‡ : not present on the Human Gene 1.0ST.

*Hs: Homo sapiens.

¤ Mm: Mus musculus.

∧ The probes on the Human Gene 1.0ST arrays recognize more than one transcript.

### Identification of biological relevant miR-375 targets using clinical CRC samples

To identify biologically relevant miR-375 targets, we set out to analyze the correlation between the expression of the above identified putative miR-375 targets and miR-375 in clinical CRC samples (normal colon mucosa n = 10 and adenocarcinoma n = 11). In all, 224 genes had at least one 7mer-m8, 7mer-m1 or 8mer miR-375 seed match in their 3′UTR and were down-regulated (FC_(log2)_≤−0.5 and p<0.05) upon miR-375 ectopic expression. Of these, 18 genes were significantly up-regulated in CRC compared to normal mucosa and showed a negative correlation to miR-375 (Pearson≤−0.6) (Table S7 in File S1). Most strikingly, YAP1 was found to be negatively correlated to miR-375 in CRC tissue samples indicating that targeting of YAP1 by miR-375 is also relevant for the tumorigenesis of colorectal cancer (Table 3 and Table S7 in File S1). Recently, a YAP1 containing transcription factor complex has been shown to positively regulate the anti-apoptotic genes BIRC5 (Survivin) and BCL2L1 [48]. Interestingly, like YAP1 we found that BIRC5 and BCL2L1 were down-regulated as a result of miR-375 up-regulation in HCT116 (FC_(log2)_: −0.9 (BIRC5) and −0.7 (BCL2L1) and p<0.05). Furthermore both BIRC5 and BCL2L1 were negatively correlated to miR-375 in clinical samples (Pearson = −0.6). These results indicate that miR-375 may act as an upstream regulator of BIRC5 and BCL2L1 through the targeting of YAP1. Stimulated by the observation that our *in silico/in vitro* approach accurately identified many known direct miR-375 targets, we decided to investigate if YAP1 and two other miR-375 target candidates HELLS and NOLC1 were directly regulated by miR-375 in CRC cells. Initial analysis confirmed the down-regulation of HELLS, NOLC1 and YAP1 at the mRNA and protein level in response to ectopic miR-375 expression in HCT116 cells (Figures 4D–F). The reduction was 40–50% both at the mRNA and protein level. In addition, down-regulation of the YAP1 downstream targets BIRC5 (54%) and BCL2L1 (72%) as a result of miR-375 ectopic expression was also confirmed at the RNA level (Figure 4E). YAP1 has previously been shown to be a direct miR-375 target in liver cells using a Luciferase reporter assay [49]. To add evidence for the direct interaction of miR-375 and YAP1 in CRC cells, we performed Ago2 immunoprecipitation (Ago2-IP) using lysates from miR-375 and Scr transfected HCT116 cells followed by YAP1 expression analysis of immunoprecipitated RNA. These analyses clearly demonstrate an Ago2 dependent immunoprecipitation of YAP1 and showed that the amount of immunoprecipitated YAP1 is enriched in the miR-375 transfected cells compared to Scr transfected cells (Figures 4G (input) and H (IP)). The expression of miR-375 in the input and IP fractions is shown in Figures S8A (input) and B (IP). The Ago2-IP analysis provided strong evidence that YAP1 is indeed a direct miR-375 target in CRC cells. We next sought to elucidate whether HELLS and NOLC1 were directly or indirectly targeted by miR-375. Luciferase reporter assay using HELLS and NOLC1 3′UTRs and a miR-375 mimic demonstrated no binding of miR-375 to the wt 3′UTR of HELLS and NOLC1 (data not shown). These results indicate that HELLS and NOLC1are probably downstream targets in the cellular pathways affected by miR-375.

### YAP1 knock-down affects the expression BIRC5 and BCL2L1 and mimics the phenotypes induced by miR-375

**Figure 5:**
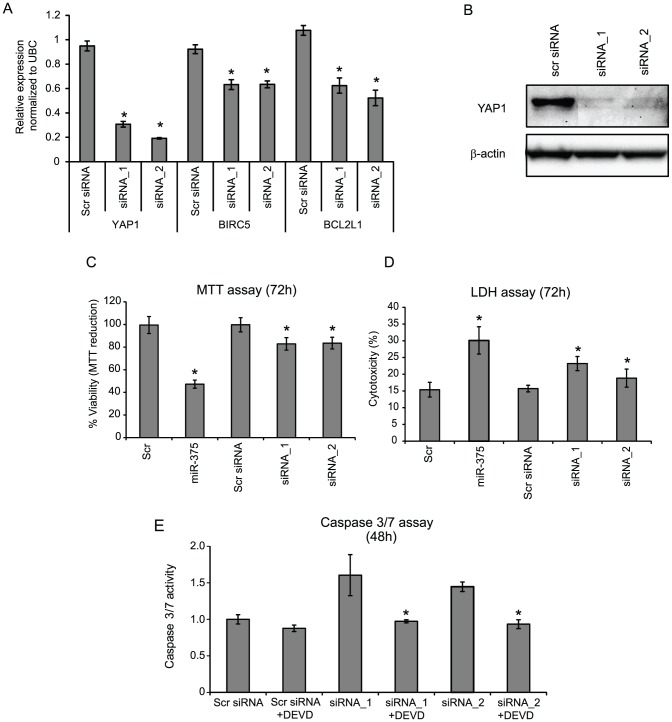
siRNA knock-down of YAP1 reduces both mRNA and protein expression in HCT116 cells. (A) The relative expression of YAP1, BCL2L1 and BIRC5 mRNA in cells transfected with YAP1 siRNA_1 or siRNA_2. The columns represent the mean of 3 replicates ± sd. *p-value<0.05 when compared to Scr. (B) Western blot demonstrating the effect of YAP1 knock-down at the protein level. Loading control: β-actin. (C) The effect of YAP1 knock-down on the viability CRC cells (MTT assay). Data are presented as ±sd. of at least 3 independent experiments each with three biological replicates and normalized to Scr. *p-value<0.05 and MTT reduction >20%. (D) Cellular death (LDH release assay): The cellular death was expressed as percentage of released LDH out of total cellular LDH. At least two independent experiments were carried out and performed in triplicates. The result of one representative experiments ±sd. is shown. *p-value<0.05. (E) Induction of apoptosis (Caspase 3/7 activity): The Caspase 3/7 activity in the lysate of siRNA transfected cells was examined by fluorometric kinetic analysis and expressed relative to the Caspase 3/7 activity in “Scr” transfected cells. Z-DEVD-fmk (DEVD) was added to the cells six hours post-transfection. Data are presented as ±sd. of at least 2 independent experiments each with three biological replicates. *p-value<0.05.

To further analyze the role of YAP1 down-regulation in the phenotypes induced by miR-375 ectopic expression, YAP1 was silenced in HCT116 cells using two different siRNAs. The knock-down efficiency of the siRNAs was more than 70% both at the mRNA and protein level (Figures 5A and B). Furthermore, like miR-375 ectopic expression, silencing of YAP1 resulted in down-regulation of BIRC5 (∼30%) and BCL2L1 (∼40–50%) further emphasizing the role of miR-375 and YAP1 in the regulation of these molecules in HCT116 cells (Figure 5A). Next we analyzed whether the silencing of YAP1could reduce the viability of HCT116 cells through induction of apoptosis thus mimicking the phenotype induced by miR-375. Indeed, elimination of YAP1 significantly reduced the viability and induced Caspase 3/7 dependent apoptotic death in HCT116 cells (Figures 5C–E). The phenotype induced by the miR-375 mimic was more pronounced than that induced by the YAP1 siRNAs, probably reflecting that YAP1 only represents one out of a number of critical nodes in the pleiotropic miR-375 network. We conclude that down-regulation of YAP1 mimics the apoptotic phenotype induced by miR-375 ectopic expression.

### Knock-down of HELLS and NOLC1 partly mimic the phenotype induce by miR-375

**Figure 6:**
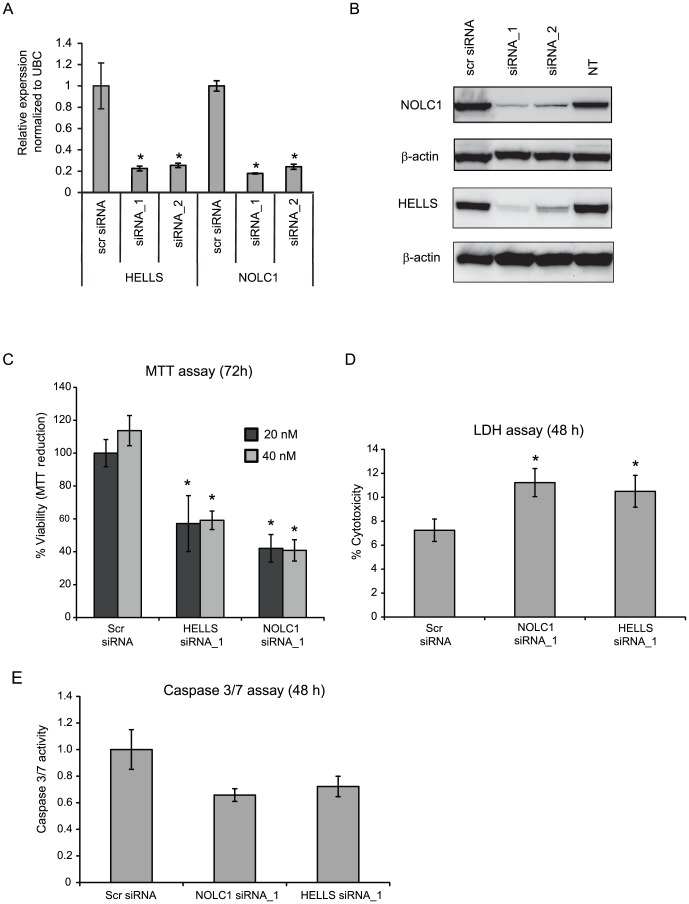
Individual knock-down of HELLS and NOLC1 reduces both mRNA and protein expression in HCT116 cells. (A) The relative expression of HELLS and NOLC1 mRNA in cells transfected with siRNA_1 and siRNA_2. The columns represent the mean of 3 replicates ± sd. (B) Western blots demonstrating the effect of siRNA target knock-down at the protein level. Loading control: β-actin. (C) The effect of HELLS and NOLC1 siRNA_1 on the viability CRC cells. Data are presented as ±sd. of at least 3 independent experiments each with three biological replicates and normalized to Scr. *p-value<0.05 and MTT reduction >20%. (D) Cellular death (LDH release assay): The cellular death was expressed as percentage of released LDH out of total cellular LDH. At least two independent experiments were carried out and performed in triplicates. The result of one representative experiments ±sd. is shown. *p-value<0.05. (E) Induction of apoptosis (Caspase 3/7 activity): The Caspase 3/7 activity in the lysate of siRNA transfected cells was examined by fluorometric kinetic analysis and expressed relative to the Caspase 3/7 activity in “Scr” transfected cells. Data are presented as ±sd. of at least 2 independent experiments each with three biological replicates. *p-value<0.05. A Scr siRNA was included as negative controls in all assays.

To investigate whether the miR-375 induced repression of HELLS and NOLC1 play a role in the phenotype induced by miR-375, we specifically silenced these mRNAs in HCT116 cells using two independent siRNAs to each target. The knock-down efficiency of the siRNAs was 70–80% at the protein level and at the mRNA level (Figures 6A and B). Next we analyzed whether knock-down of HELLS and NOLC1 could reduce the viability of HCT116 and induce a pro-apoptotic phenotype, thus mimicking the phenotype induced by miR-375. Indeed, individual elimination of HELLS and NOLCL1 reduced the viability and induced cellular death in a manner similar to miR-375 ectopic expression (Figures 6C and D) but did not induce apoptotic death (Figure 6E) and hence other miR-375 targets such as YAP1 are responsible for the apoptotic phenotype. In conclusion, reduced expression of HELLS and NOLC1 only partly mimic the phenotypes induced by miR-375

### Characterization of stable HCT116 with inducible miR-375 expression and *in vivo* tumor growth

**Figure 7:**
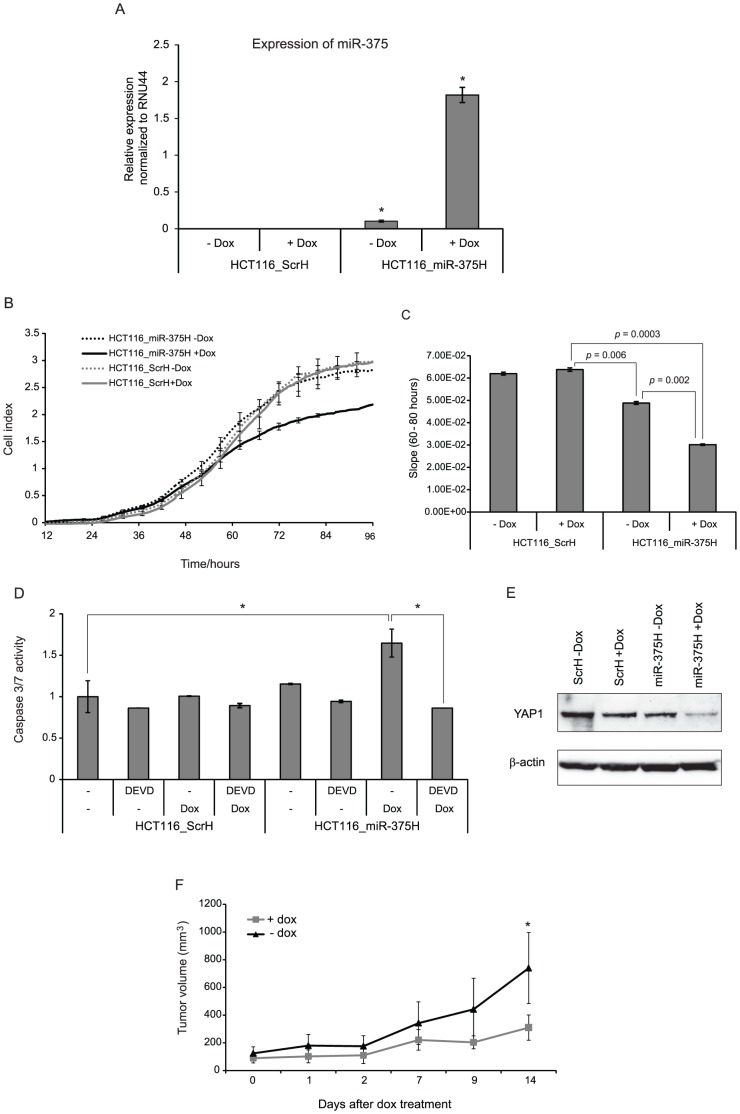
Generation and characterization of stable HCT116 cells with inducible miR-375 expression (HCT116-miR-375H). (A) Dox treatment induces increased expression of miR-375 in HCT116_miR-375H cells. The Relative expression of miR-375 was measured using RT-qPCR. The columns represent the mean of 3 replicates ± sd.*p-value<0.05 when compared to HCT116_ScrH cells. (B) xCELLigence real-time monitoring of cell proliferation. The cell index from time 12–96 hours is shown. Dox was added at time 0. (C) Dox treatment significantly reduces the proliferation of HCT116_miR-375H cells. Following the real-time monitoring in B, the slope (rate of changes in cell index) was calculated from time 60–80 hours (i.e. when changes in cell viability were apparent) and presented graphically. (D) Dox treatment specifically induces Caspase 3/7 dependent apoptosis in HCT116 miR-375H cells. The Caspase 3/7 activity was examined by fluorometric kinetic analysis and expressed relative to the Caspase 3/7 activity in untreated HCT116_ScrH cells. Z-DEVD-fmk (DEVD) was added to the cells six hours post-transfection. Data are presented as ±sd. of at least 2 independent experiments each with three biological replicates. *p-value<0.05. (E) Western blotting demonstrating down-regulation of YAP1 in dox treated HCT116_miR-375H cells compared to non-treated and HCT116_ScrH cells. Loading control: β-actin. (F) miR-375 expression reduces tumor growth *in vivo*. Growth curves of tumors generated in nude mice injected with HCT116_miR-375H cells treated with (n = 4) or without (n = 4) dox in the drinking water. Dox was added to the drinking water when the tumor size was >50 mm^3^. Data marks and bars represent the mean ±sd. *p-value<0.05.

To analyze the effect of miR-375 on tumor growth *in vivo* HCT116 cells stably transfected with a polycistronic dox-inducible expression cassette, producing both tRFP and miR-375, was generated. The initial pool of stably transfected cells exhibited varying levels of tRFP upon dox induction (data not shown). In order to obtain a pool of cells with a more uniform expression of tRFP, and thus miR-375, upon dox induction, we applied FACS sorting. Cells with tRFP expression levels 100–1000 times above the background level in untreated cells (HCT116_miR-375H and HCT116_ScrH) were isolated and used in all subsequent analyses. Compared to untreated HCT116_miR375H cells dox treated cells induced an eighteen-fold increase in miR-375 expression level. While no miR-375 expression was observed in the HCT116_ScrH cells +/− dox, a minor level of miR-375 expression was observed in the untreated HCT116_miR-375H cells (Figure 7A), which could indicate a minor leakage from the miR-375-tRFP expression cassette in the absence of dox. However, the phenotypic characterization of HCT116_miR-375H cells clearly showed that dox treatment significantly reduced the cell proliferation and induced Caspase 3/7 dependent apoptosis in the HCT116_miR-375H cells when compared to HCT116-ScrH cells or untreated HCT116-miR-375H cells (Figures 7B–D). In addition, the YAP1 protein was specifically down-regulated in dox treated HCT116_miR-375H cells (Figure 7E). *In vivo* tumor growth was analyzed in nude mice by subcutaneous injection of HCT116_miR-375H cells. The growth of tumors in dox treated mice was compared to the growth in untreated animals. In accordance with the *in vitro* results, the results of the *in vivo* analysis clearly indicate that miR-375 expression reduces tumor growth (Figure 7F). All together, the above results strongly indicate that miR-375 has the ability to suppress tumor growth through the induction of apoptosis.

## Discussion

Traditional expression profiling of miRNA in cancer tissues identifies differentially expressed miRNAs which may act either as “passenger” or “drivers” in tumorigenesis. Functional screening on the contrary identifies miRNAs that result in cancer associated phenotypes likely to act as “driver” miRNAs. However the functional assays are performed in cell lines with the risk of identifying candidate miRNAs that are not important for tumor development *in vivo*. In the present study we have combined the miRNA expression profiling in CRC tissue samples with functional screening thereby making it possible to identify clinically relevant miRNAs and miRNA targets in CRC, which may eventually be used as biomarkers or therapeutic targets.

High-throughput functional screening of miRNAs has previously been carried out in cell lines originating from testicular germ cell tumors, pancreatic cancer and CRC [17]–[20]. Most of these studies were performed using only one cell line and only Cekaite et al. related their findings to the expression of the miRNAs in clinical samples [18]. In the present study the functional screening was performed in six different CRC cell lines allowing us to identify both general mechanisms in colorectal cancer and cell lines specific effects. Our results clearly demonstrate the importance of combing the functional screening with profiling of tissue samples since many of the Top-40 ranked miRNAs were either not differentially expressed or not expressed in CRC tissue samples. In accordance with this only one out of 23 miRNAs (miR-483-5p) identified in a previous functional screen using the CRC cell line DLD1 [17] was significantly dys-regulated in the CRC samples in the present study. Furthermore, some of the miRNAs differentially expressed in clinical samples only exhibited a phenotype in one of the cell lines analyzed and would have been missed if we had not run a panel of cell lines.

**Figure 8:**
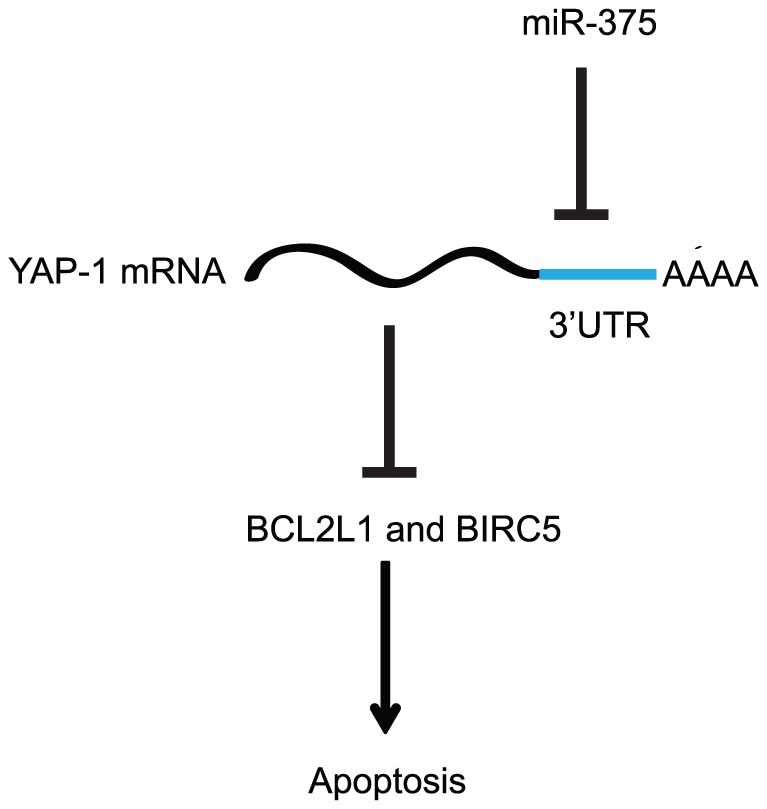
Model of the role of miR-375 in the regulation of apoptotic death.

We selected miR-375 for detailed functional characterization and target identification. In addition to the results of the present study, down-regulation of miR-375 has been demonstrated in several types of cancer including CRC [35], [40], [49]–[51]. The mechanism behind miR-375 down-regulation has been studied in several cancers. Originally, *MIR-375* hypermethylation was reported in cell lines originating from breast and gastric cancer [38], [52]. Methylation analysis in melanoma and esophageal cancer later confirmed that methylation played a role in miR-375 down-regulation not only in cell lines but also in tissue samples [37], [40], [42]. In CRC *MIR-375* promoter methylation has only been demonstrated in the cell line HCT116 [39]. Additionally, miR-375 has also been shown to be up-regulated upon 5-aza-2′-deoxycytidine treatment in HCT116 and to a less extent in DLD1 [53]. Thorough analysis of *MIR-375* methylation and expression in CRC cell lines and tissue samples only identified *MIR-375* promoter methylation and concurrent miR-375 down-regulation in three CRC cell lines including HCT116. None of the tissue samples demonstrated *MIR-375* promoter methylation although miR-375 was clearly down-regulated in a subset of the samples indicating that *in vivo* miR-375 is mainly regulated by other mechanisms than hypermethylation in CRCs. We cannot rule out that methylation of other CpG sites than the ones addressed in the present study are important for miR-375 down-regulation in CRC, however, previous methylation analysis of *MiR-375* has demonstrated homogenous methylation throughout the analyzed genomic regions, making this unlikely. Alternatively, it has been suggested that miR-375 might be regulated negatively by the Wnt pathway [25], [43]. The ChIP analysis carried out in the present study, however, demonstrated that miR-375 is most likely not under direct β-catenin/TCF4 control and rather suggest that yet unidentified downstream targets of the Wnt pathway affect miR-375 expression in CRC. To address the functional role of miR-375, we and others have carried out *in vitro* phenotype analyses. In the present study, miR-375 was shown to reduce viability and to induce Caspase 3/7 dependent apoptotic death in CRC cell lines. The reduction of cellular viability by miR-375 has previously been shown in cell lines from several cancers, [40], [49], [50], [52], [54] whereas the apoptotic phenotype has been demonstrated in cells from gastric and esophageal cancer [37], [52]. In addition, suppression of colony formation and reduced migration and invasion has also been linked to miR-375 expression [37], [40], [42], [49]. Recently, miR-375 was also shown to play a role in cell cycle regulation through the inhibition of G1/S transition in HCT116 cells [54]. To study the effect of miR-375 on tumor growth *in vivo* HCT116 cells stably transfected with an inducible miR-375 expression-cassette were used to generate mouse xenograft tumors. Induction of miR-375 expression significantly reduced the growth of the tumors confirming the results from esophageal squamous cell carcinoma in which miR-375 was shown to effectively suppress tumor formation and metastasis [42]. Overall, the frequent down regulation of miR-375 in cancer and the phenotypic characterization of miR-375 *in vivo* and *in vitro* clearly emphasize its tumor suppressive role and have encouraged the search for miR-375 targets mediating the tumor suppressive effects. At present, YWHAZ, JAK2, PDK1 and YAP1 have been identified as cancer relevant direct miR-375 targets in human gastric, esophageal and liver cancer using Luciferase reporter assays [37], [49], [50], [52], [55] . Among others, YAP1 is up-regulated in many epithelial cancers, and is mainly known as an effector of the Hippo signaling pathway involved in cell growth, division and apoptosis [56], [57], whereas up-regulation of YWHAZ has been shown to be associated with low miR-375 expression and reduced overall survival in gastric cancer [58]. In the present study, only YAP1 demonstrated an inverse correlation with the expression of miR-375 both *in vitro* and in clinical CRC samples. Additionally, knockdown of YAP1 using siRNAs mimicked the apoptotic phenotype induced by miR-375. To study the binding of miR-375 to YAP1 in a physiologically relevant manner we carried out Ago2-IP. These analyses showed that YAP1 is enriched in immunoprecipitates from miR-375 transfected cells compared to Scr transfected cells in an Ago2 dependent manner, thus providing strong evidence that YAP1 is indeed a direct miR-375 target in CRC cells. Recently, YAP1 has been suggested to be part of a complex required for survival of β-catenin driven cancers including CRC [48]. A complex consisting of the transcription factor TBX5, YAP1, β-catenin and YES1 was found to regulate the expression of the anti-apoptotic genes BIRC5 and BCL2L1. Interestingly, both BIRC5 and BCL2L1 were up-regulated in CRC tissue samples and down-regulated as a result of miR-375 up-regulation or siRNA silencing of YAP1 in HCT116 in the present study. Thus down-regulation of BIRC5 and BCL2L1 may among others explain the apoptotic phenotype induced as a result of miR-375 ectopic expression. We hypothesize that miR-375 exerts its tumor suppressive role partly by acting as an upstream regulator of BIRC5 and BCL2L1 through the targeting of YAP1 (Figure 8). miR-375 has also been shown to inhibit G1/S transition in HCT116 [54]. In the present study, we identified several genes related to cell cycle progression among the clinically relevant putative miR-375 targets, including HELLS and NOLC1. HELLS is overexpressed in many human tumors and has been shown to hinder cell cycle re-entry and cellular growth whereas NOLC1 expression is crucial for the growth of nasopharyngeal carcinomas [59], [60]. We showed that HELLS and NOLC1 were down-regulated both at the mRNA and protein level as a result of miR-375 ectopic expression. Additionally, their knock-down reduced cell viability and induced cellular death mimicking the phenotype induced by miR-375, although they were not identified as direct miR-375 targets using a Luciferase reporter assay.

In conclusion, combing high-throughput functional screening with miRNA profiling of CRC tissue samples, we have identified clinically relevant miRNAs in colorectal cancer including miR-375. We showed that methylation of miR-375 is not a common mechanism behind miR-375 down-regulation in CRC. Finally, miR-375 target analysis demonstrated that the pro-apoptotic role of miR-375 by may be exerted through direct targeting of YAP1 resulting in down-regulation of the anti-apoptotic genes *BIRC5* and *BCL2L1*.

## Supporting Information

Figure S1 **Line graph distributions of the cPARP and Ki67 z-scores for each pre-miR in six CRC cells lines.** Selected miRNAs are shown. *Pre-mir negative control #2.
(PDF)

Figure S2 **Cellular viability (MTT assay) upon ectopic expression of the miRNAs.** Data are presented as ±sd. of at least 3 independent experiments each with three biological replicates and normalized to Scr. *p-value<0.05 and MTT reduction >20%. The pre-miR-145 and Scr were included as positive and negative controls respectively. Non treated cells (NT) and Lipofectamine only (Lipo).
(PDF)

Figure S3 **Cellular death (LDH release assay and Caspase 3/7 activity as a result of ectopic miRNA expression.** The cellular death (left column) was expressed as percentage of released LDH out of total cellular LDH. At least two independent experiments were carried out and performed in triplicates. The results of one representative experiment ±sd. of the triplicates are shown.The Caspase 3/7 activity (rigth column) in the lysate of pre-miRNA transfected cells was examined by fluorometric kinetic analysis and expressed relative to the Caspase 3/7 activity in “scr” transfected cells. Data are presented as ±sd. of at least 2 independent experiments each with three biological replicates. *p-value<0.05. Non-treated cells (NT), Lipofectamine only (Lipo) and Scr (negative control).
(PDF)

Figure S4 **The expression of miR-9 and miR-138 in laser capture microdissected colorectal cancer tissue.** The expression was analyzed in epithelial and stromal cells from 3 paired colorectal adenocarcinomas and adjacent normal colon mucosa using RT-qPCR. The columns represent the mean expression in three samples ± sd.
(PDF)

Figure S5 **miR-375 expression in cohort 2.** (A) Box plots comparing the relative expression of miR-375 in 25 samples from normal colon mucosa and 63 primary MSS stage I–IV CRCs (T2-4, N0-3, M0/1)×Minimum and maximum outliers. (B) The miR-375 expression in individual samples. The expression was measured in triplicates using RT-qPCR and normalized to RNU44. The red bars shows the mean in the normal mucosa (N)(mean = 4.7) and the tumors (T)(mean = 1.0).
(PDF)

Figure S6 **Association of TCF4 with chromatin in the genomic region of miR-375 using ChIP followed by qPCR.** MYC 3′enhancer region (3′enh) = positive control region and Myo ex2 = negative control region. The CD58 antibody was used as a negative control.
(PDF)

Figure S7 **Ingenuity pathway analysis of genes affected by miR-375 over-expression.** Biological functions significantly associated with altered intracellular levels of miR-375 in HCT116 cells 48 h post transfection. Only mRNAs demonstrating p-values<0.05 and log2 ratios <−0.5 or >0.5 (1236 genes) (A) or <−1.0 or >1.0 (206 genes) (B), comparing miR-375 and Scr transfected cells, were included in the analyses. Threshold: p = 0.05. The analyses were based on knowledge from all species, all tissues, and all data sources.
(PDF)

Figure S8 **Ago2 immunoprecipitation from cell lysates of miR-375 or Scr transfected cells.** (A) RT-qPCR expression analysis of miR-375 in the cell lysates (input) used for Ago2 immunoprecipitation. (B) Ago2 immuneprecipitation from the cell lysates (IP) followed by miR-375 expression analysis using RT-qPCR. Immunoprecipitation with a FLAG antibody was used as negative control. A 1∶1 ratio of the lysates from miR-375 and Scr transfected cells was used for FLAG immunoprecipitation. The columns represent the mean of 3 replicates ± sd.
(PDF)

Methods S1 **Supplementary Material.**
(DOCX)

File S1 **Tables S1–S3, S6 and S7.**
(DOCX)

File S2 **Tables S4 and S5.**
(DOCX)
