# Supplementary material for: Functional Screening Identifies miRNAs Influencing Apoptosis and Proliferation in Colorectal Cancer
Source: PLoS One. 2014 Jun 3;9(6):e96767. doi: 10.1371/journal.pone.0096767 (PMC4043686; doi:10.1371/journal.pone.0096767)
Supplement: Figure S1 — Line graph distributions of the cPARP and Ki67 z-scores for each pre-miR in six CRC cells lines. Selected miRNAs are shown. *Pre-mir negative control #2. (PDF) [file pone.0096767.s001.pdf]

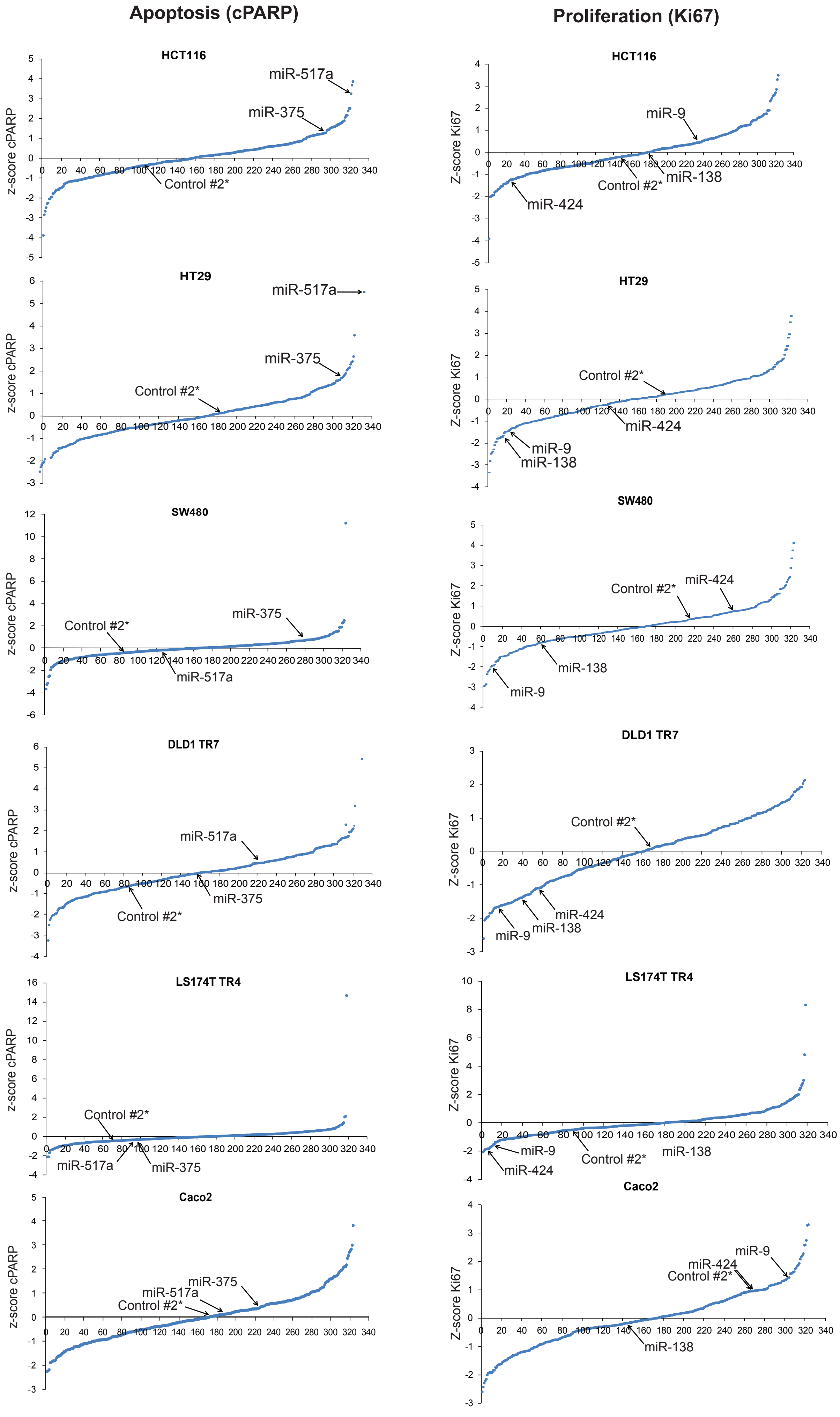

Supplementary Fig. S1. Line graph distributions of the cPARP and Ki67 z-scores for each pre-miR in six CRC cells lines. Selected miRNAs are shown.*Pre-mir negative control #2
