# Supplementary material for: Functional Screening Identifies miRNAs Influencing Apoptosis and Proliferation in Colorectal Cancer
Source: PLoS One. 2014 Jun 3;9(6):e96767. doi: 10.1371/journal.pone.0096767 (PMC4043686; doi:10.1371/journal.pone.0096767)
Supplement: Figure S2 — Cellular viability (MTT assay) upon ectopic expression of the miRNAs. Data are presented as ±sd. of at least 3 independent experiments each with three biological replicates and normalized to Scr. *p-value<0.05 and MTT reduction >20%. The pre-miR-145 and Scr were included as positive and negative controls respectively. Non treated cells (NT) and Lipofectamine only (Lipo). (PDF) [file pone.0096767.s002.pdf]

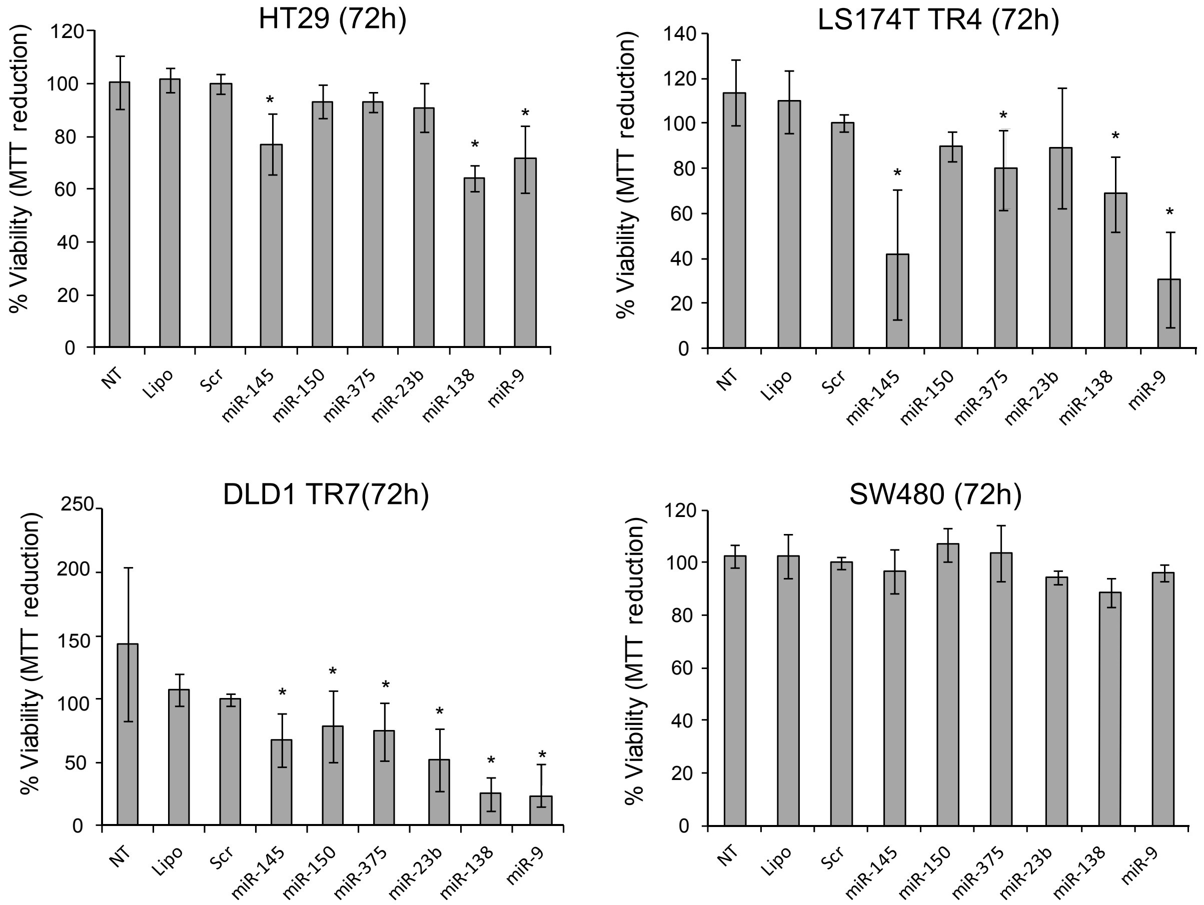

Supplementary Fig. S2. Cellular viability (MTT assay) upon ectopic expression of the miRNAs. Data are presented as ±sd. of at least 3 independent experiments each with three biological replicates and normalized to Scr. *p-value <0.05 and MTT reduction >20%. The pre-miR-145 and Scr were included as positive and negative controls respectively. Non treated cells (NT) and Lipofectamine only (Lipo).
