# Supplementary material for: Functional Screening Identifies miRNAs Influencing Apoptosis and Proliferation in Colorectal Cancer
Source: PLoS One. 2014 Jun 3;9(6):e96767. doi: 10.1371/journal.pone.0096767 (PMC4043686; doi:10.1371/journal.pone.0096767)
Supplement: Figure S3 — Cellular death (LDH release assay and Caspase 3/7 activity as a result of ectopic miRNA expression. The cellular death (left column) was expressed as percentage of released LDH out of total cellular LDH. At least two independent experiments were carried out and performed in triplicates. The results of one representative experiment ±sd. of the triplicates are shown.The Caspase 3/7 activity (rigth column) in the lysate of pre-miRNA transfected cells was examined by fluorometric kinetic analysis and expressed relative to the Caspase 3/7 activity in “scr” transfected cells. Data are presented as ±sd. of at least 2 independent experiments each with three biological replicates. *p-value<0.05. Non-treated cells (NT), Lipofectamine only (Lipo) and Scr (negative control). (PDF) [file pone.0096767.s003.pdf]

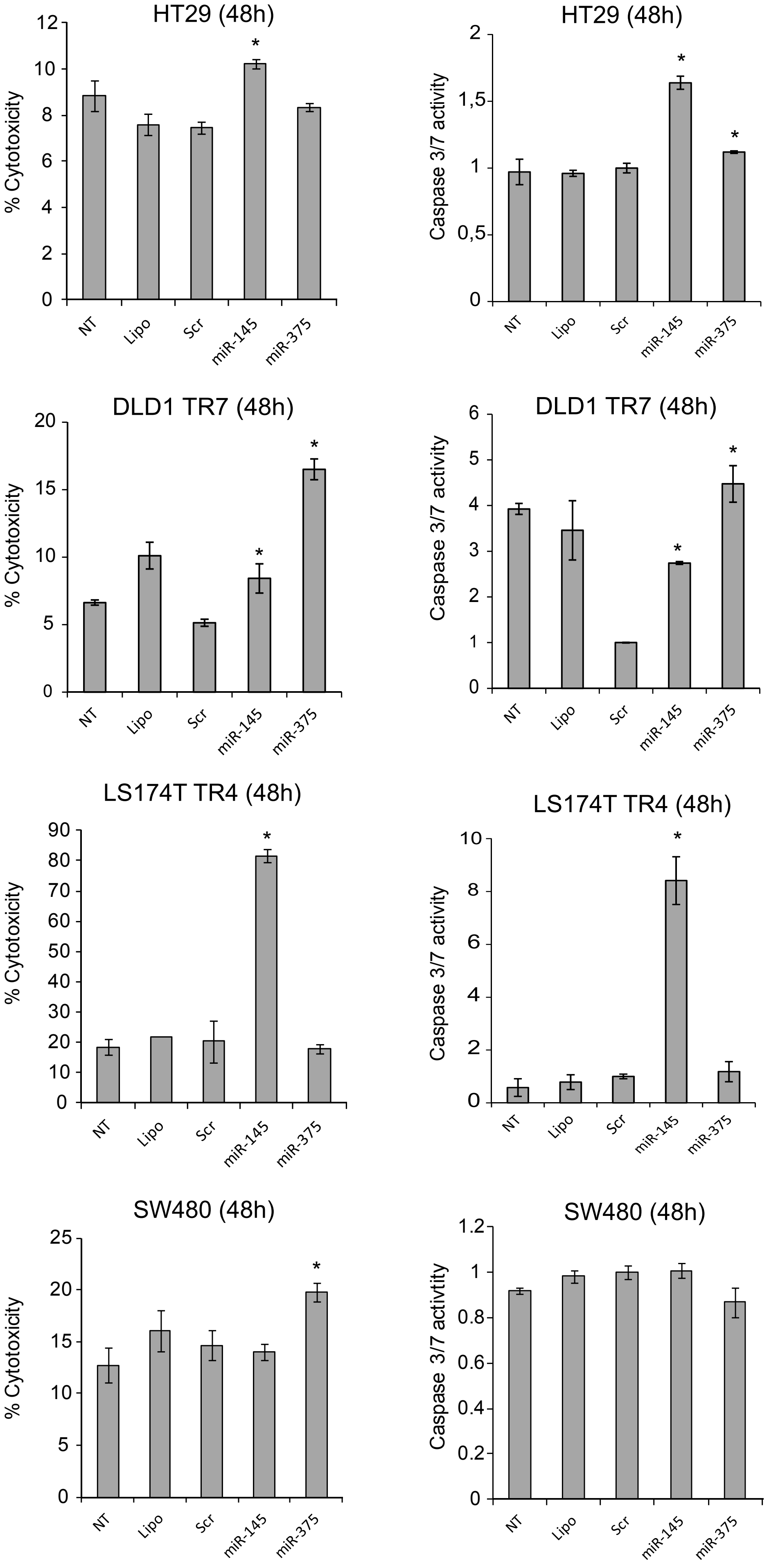

Supplementary Fig. S3. Cellular death (LDH release assay and Caspase 3/7 activity as a result of ectopic miRNA expression. The cellular death (left column) was expressed as percentage of released LDH out of total cellular LDH. At least two independent experiments were carried out and performed in triplicates. The results of one representative experiment ±sd. of the triplicates are shown.The Caspase 3/7 activity (rigth column) in the lysate of pre-miRNA transfected cells was examined by fluorometric kinetic analysis and expressed relative to the Caspase 3/7 activity in “scr” transfected cells. Data are presented as ±sd. of at least 2 independent experiments each with three biological replicates. *p-value <0.05. Non-treated cells (NT), Lipofectamine only (Lipo) and Scr (negative control).
