# Supplementary material for: Functional Screening Identifies miRNAs Influencing Apoptosis and Proliferation in Colorectal Cancer
Source: PLoS One. 2014 Jun 3;9(6):e96767. doi: 10.1371/journal.pone.0096767 (PMC4043686; doi:10.1371/journal.pone.0096767)
Supplement: Figure S4 — The expression of miR-9 and miR-138 in laser capture microdissected colorectal cancer tissue. The expression was analyzed in epithelial and stromal cells from 3 paired colorectal adenocarcinomas and adjacent normal colon mucosa using RT-qPCR. The columns represent the mean expression in three samples ± sd. (PDF) [file pone.0096767.s004.pdf]

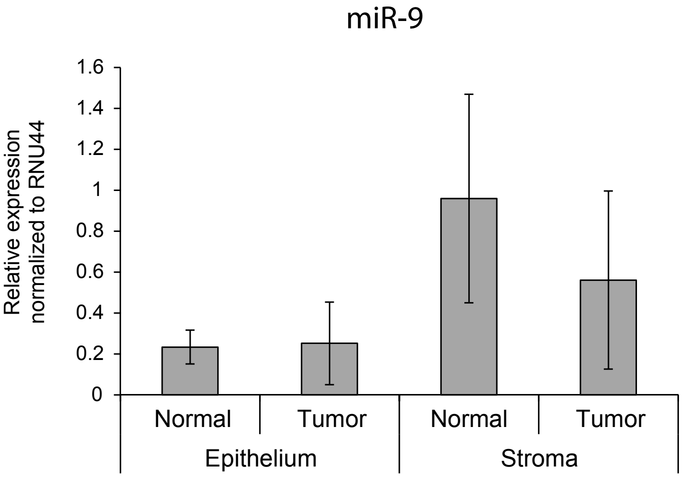

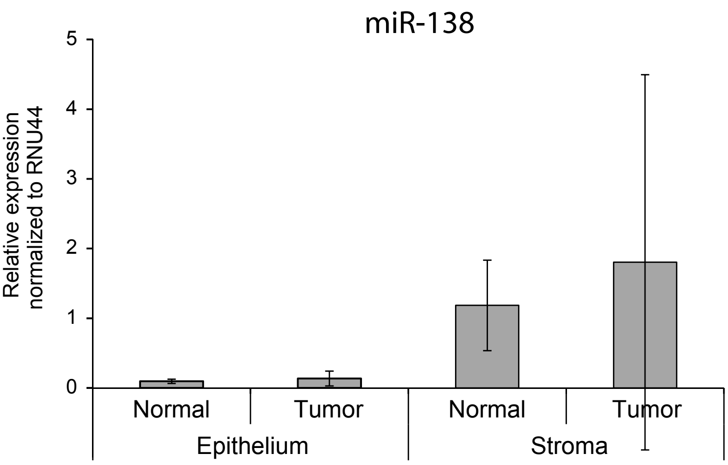

Supplementary Fig. S4. The expression of miR-9 and miR-138 in laser capture microdissected colorectal cancer tissue. The expression was analyzed in epithelial and stromal cells from 3 paired colorectal adenocarcinomas and adjacent normal colon mucosa using RT-qPCR. The columns represent the mean expression in three samples ± sd.
