# Supplementary material for: Functional Screening Identifies miRNAs Influencing Apoptosis and Proliferation in Colorectal Cancer
Source: PLoS One. 2014 Jun 3;9(6):e96767. doi: 10.1371/journal.pone.0096767 (PMC4043686; doi:10.1371/journal.pone.0096767)
Supplement: Figure S5 — miR-375 expression in cohort 2. (A) Box plots comparing the relative expression of miR-375 in 25 samples from normal colon mucosa and 63 primary MSS stage I–IV CRCs (T2-4, N0-3, M0/1)×Minimum and maximum outliers. (B) The miR-375 expression in individual samples. The expression was measured in triplicates using RT-qPCR and normalized to RNU44. The red bars shows the mean in the normal mucosa (N)(mean = 4.7) and the tumors (T)(mean = 1.0). (PDF) [file pone.0096767.s005.pdf]

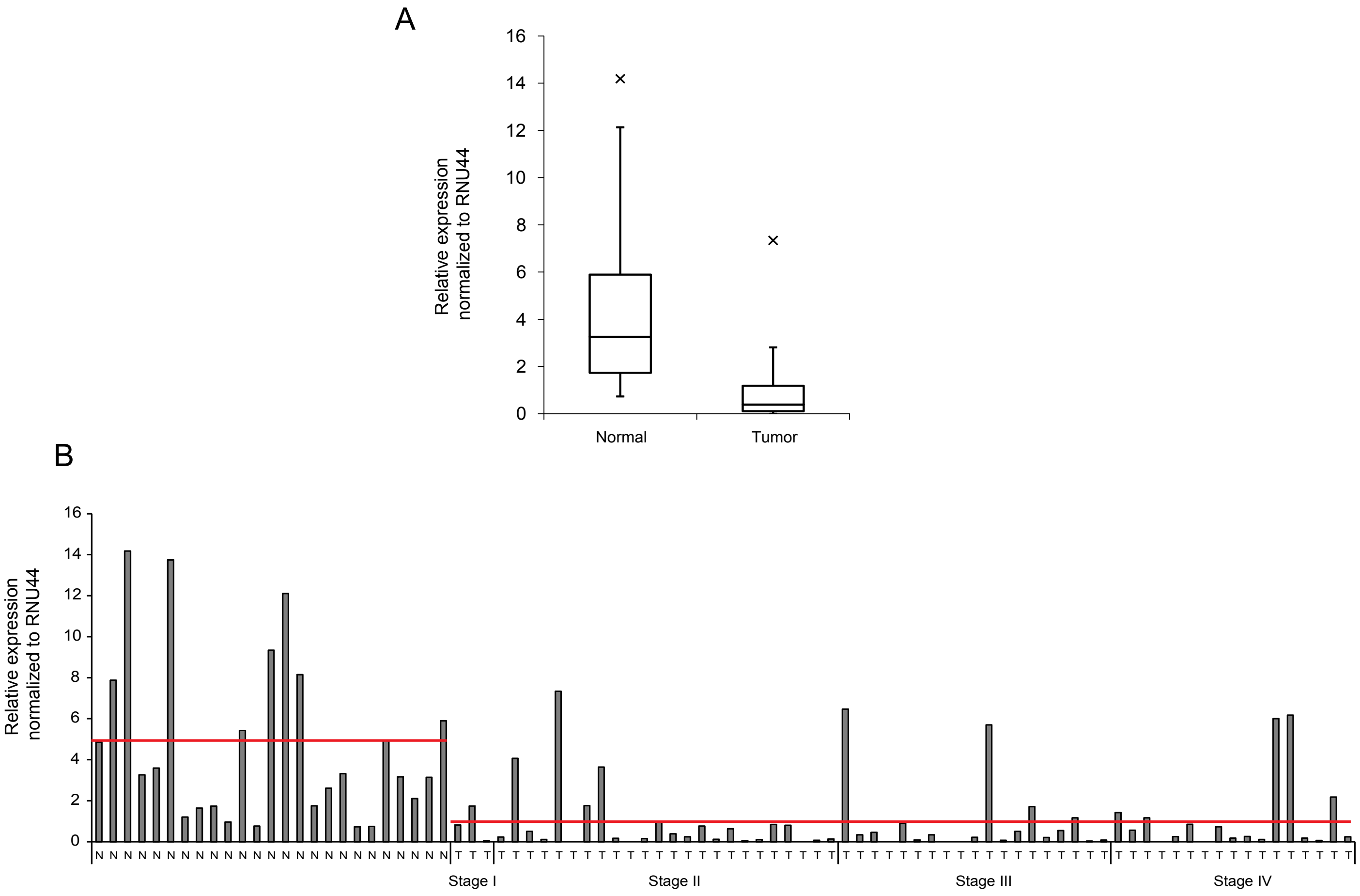

Supplementary Fig. S5. miR-375 expression in cohort 2. (A) Box plots comparing the relative expression of miR-375 in 25 samples from normal colon mucosa and 63 primary MSS stage I-IV CRCs (T2-4, N0-3, M0/1) × Minimum and maximum outliers. (B) The miR-375 expression in individual samples. The expression was measured in triplicates using RT-qPCR and normalized to RNU44. The red bars shows the mean in the normal mucosa (N)(mean = 4.7) and the tumors (T)(mean = 1.0).
