# Supplementary material for: Functional Screening Identifies miRNAs Influencing Apoptosis and Proliferation in Colorectal Cancer
Source: PLoS One. 2014 Jun 3;9(6):e96767. doi: 10.1371/journal.pone.0096767 (PMC4043686; doi:10.1371/journal.pone.0096767)
Supplement: Figure S6 — Association of TCF4 with chromatin in the genomic region of miR-375 using ChIP followed by qPCR. MYC 3′enhancer region (3′enh) = positive control region and Myo ex2 = negative control region. The CD58 antibody was used as a negative control. (PDF) [file pone.0096767.s006.pdf]

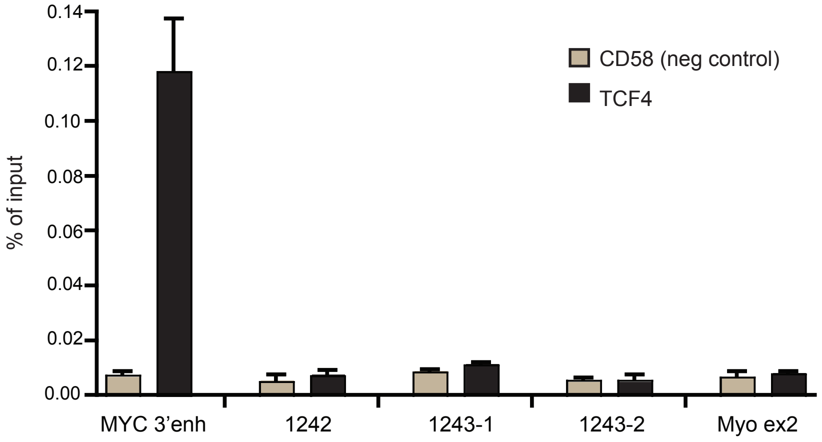

Supplementary Fig. S6. Association of TCF4 with chromatin in the genomic region of miR-375 using ChIP followed by qPCR. MYC 3’enhancer region (3’enh) = positive control region and Myo ex2 = negative control region. The CD58 antibody was used as a negative control.
