# Supplementary material for: Functional Screening Identifies miRNAs Influencing Apoptosis and Proliferation in Colorectal Cancer
Source: PLoS One. 2014 Jun 3;9(6):e96767. doi: 10.1371/journal.pone.0096767 (PMC4043686; doi:10.1371/journal.pone.0096767)
Supplement: Figure S7 — Ingenuity pathway analysis of genes affected by miR-375 over-expression. Biological functions significantly associated with altered intracellular levels of miR-375 in HCT116 cells 48 h post transfection. Only mRNAs demonstrating p-values<0.05 and log2 ratios <−0.5 or >0.5 (1236 genes) (A) or <−1.0 or >1.0 (206 genes) (B), comparing miR-375 and Scr transfected cells, were included in the analyses. Threshold: p = 0.05. The analyses were based on knowledge from all species, all tissues, and all data sources. (PDF) [file pone.0096767.s007.pdf]

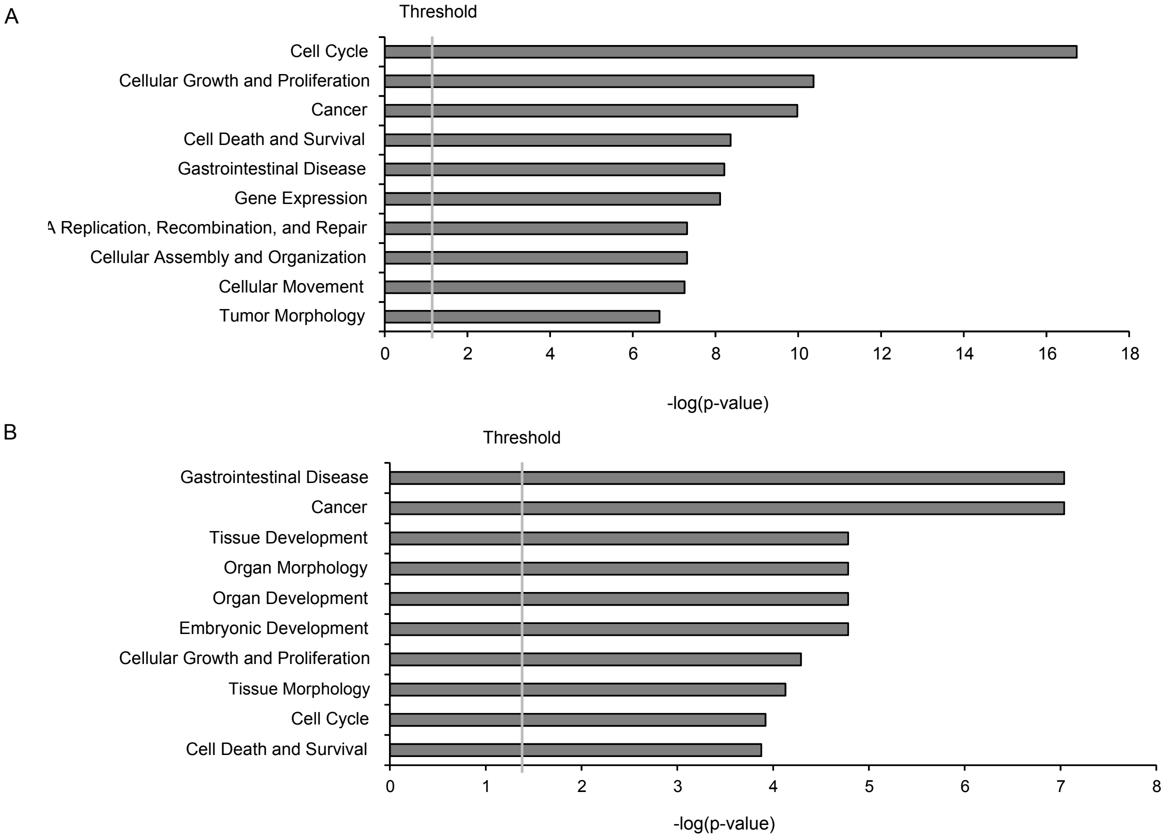

Supplementary Fig. S7. Ingenuity pathway analysis of genes affected by miR-375 over-expression. Biological functions significantly associated with altered intracellular levels of miR-375 in HCT116 cells 48 h post transfection. Only mRNAs demonstrating p-values < 0.05 and log2 ratios < -0.5 or > 0.5 (1236 genes) (A) or < -1.0 or > 1.0 (206 genes) (B), comparing miR-375 and Scr transfected cells, were included in the analyses. Threshold: p=0.05. The analyses were based on knowledge from all species, all tissues, and all data sources.
