# Supplementary material for: Functional Screening Identifies miRNAs Influencing Apoptosis and Proliferation in Colorectal Cancer
Source: PLoS One. 2014 Jun 3;9(6):e96767. doi: 10.1371/journal.pone.0096767 (PMC4043686; doi:10.1371/journal.pone.0096767)
Supplement: Figure S8 — Ago2 immunoprecipitation from cell lysates of miR-375 or Scr transfected cells. (A) RT-qPCR expression analysis of miR-375 in the cell lysates (input) used for Ago2 immunoprecipitation. (B) Ago2 immuneprecipitation from the cell lysates (IP) followed by miR-375 expression analysis using RT-qPCR. Immunoprecipitation with a FLAG antibody was used as negative control. A 1∶1 ratio of the lysates from miR-375 and Scr transfected cells was used for FLAG immunoprecipitation. The columns represent the mean of 3 replicates ± sd. (PDF) [file pone.0096767.s008.pdf]

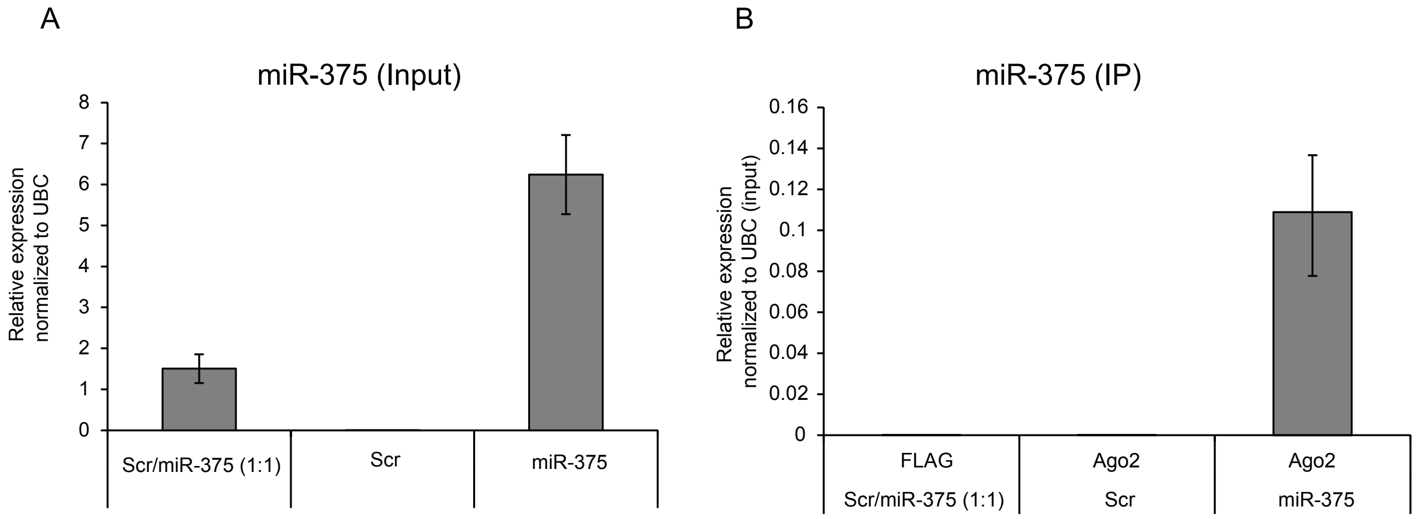

Supplementary Fig. S8. Ago2 immunoprecipitation from cell lysates of miR-375 or Scr transfected cells. (A) RT-qPCR expression analysis of miR-375 in the cell lysates (input) used for Ago2 immunoprecipitation. (B) Ago2 immuneprecipitation from the cell lysates (IP) followed by miR-375 expression analysis using RT-qPCR. Immunoprecipitation with a FLAG antibody was used as negative control. A 1:1 ratio of the lysates from miR-375 and Scr transfected cells was used for FLAG immunoprecipitation. The columns represent the mean of 3 replicates ± sd.
