# Supplementary material for: Functional Screening Identifies miRNAs Influencing Apoptosis and Proliferation in Colorectal Cancer
Source: PLoS One. 2014 Jun 3;9(6):e96767. doi: 10.1371/journal.pone.0096767 (PMC4043686; doi:10.1371/journal.pone.0096767)
Supplement: Methods S1 — Supplementary Material. (DOCX) [file pone.0096767.s009.docx]

**Supplementary material**

**Materials and Methods**

***Clinical samp*les**

A total of 46 fresh frozen microsatellite stable (MSS), primary stage II (T2-4, N0, M0) CRCs and 14 normal colon mucosa (cohort 1) were selected from the colorectal cancer biobank at the Department of Molecular Medicine, Aarhus University Hospital, Skejby, Denmark, at the Department of Genetics and Pathology Pomeranian Medical University, Szczecin, Poland, and at the Royal Melbourne Hospital and Western Hospital, Melbourne, Australia. An independent cohort consisting of 25 RNAlater (Qiagen, Hilden, Germany) preserved normal colon mucosa and 63 MSS, primary stage I-IV (T2-4, N0-3, M0/1) CRCs (cohort 2) was used for validation. The samples in cohort 2 were all from the Department of Surgery, Vejle Hospital, Denmark. Patients who had received preoperative chemotherapy and/or radiation of rectal cancers were excluded. Postoperatively the tumors were histologically classified and staged according to the pTNM system. Cases with hereditary colorectal cancer syndromes were not included in the study.

***Cell lines***

The human colon cancer cells lines; HT29, HCT116, HCT15, Colo205, DLD1, LS174T, SW620 and SW480 were purchased from the American Type Culture Collection (ATCC). The LS174T TR4 and the DLD1 TR7 cells lines were kindly provided by Hans Clevers, Hubrecht Institute, Utrecht, the Netherlands. Caco2 and HEK293T cells were kind gifts from The Research Unit for Molecular Medicine and from Pia Møller Martensen, Department of Molecular Biology, Aarhus University, Aarhus, Denmark, respectively. The HCT116 and HT29 cell lines were grown in McCoy’s 5A Media (Gibco, Invitogen Carlsbad, CA, USA). DLD1/DLD TR7 cells were grown in RPMI 1640 with Hepes whereas HCT15, Colo205, and LS174T/LS174 TR4 were grown in RPMI 1640 without Hepes (Gibco, Invitrogen). The SW620, SW480, Caco2 and HEK293T cells were cultured in D-MEM (Gibco, Invitrogen). The culture media was supplemented with 10% Fetal Calf Serum (FCS) (Gibco, Invitrogen), except for DLD1 and DLD1 TR7 cells, which were cultured in 5% FCS. The cells were cultured at 37°C in an atmosphere of 5% CO_2_. All cell lines were authenticated according to the recommendation of (American Type Tissue Collection) (ATCC, Manassas, VA, USA) using short tandem repeat (STR) profiling. The obtained STR profiles all matched those published by ATCC (data not shown).

***MiRNA functional library screen***

The cell spot microarray technology was used to generate high density pre-miRNA transfection microarrays as previously described with minor modifications[1]. Briefly, a library holding 319 synthetic human pre-miRNAs (Ambion pre-miR v1.0) (Ambion, Austin, TX, USA) was used for printing of the arrays. For each sample 5 µl of 2 µM precursor miR’s was mixed with 1.2 µl of siLentFect transfection reagent (Bio-Rad) and 2 µl of growth factor reduced Matrigel (BD Bioscience, Franklin Lakes, NJ, USA) diluted with 2 µl of OptMEM-I (Gibco, Carlsbad, CA, USA) supplemented with 250 mM sucrose. Arrays were printed on polystyrene microplates with four rectangular wells (NUNC, Penfield, NY, USA) using 200 µm solid microarraying pins. Subsequently, the CRC cells (HCT116, LS174T TR4, DLD1 TR7, HT29, Caco2 and SW480) were seeded onto the arrays and the reverse transfection was carried out for 48 hours. To allow microscopic detection of pre-miRNAs effects influencing cell proliferation and/or apoptosis the arrays were immunostained for Ki-67 (proliferation marker) and for cleaved poly ADP-ribose polymerase (cPARP) (apoptosis marker). DNA was stained using 4’,6 Diamidino-2-phenylindole (DAPI)(Invitrogen, Carlsbad, CA, USA) or SYTO60 (Invitrogen).  The microarray analysis was performed with microscopic imaging of the arrays using scanR high content imager (Olympus) and the effect of miRNA over-expression on apoptosis and cell proliferation was considered as previously described [1].

***RNA and miRNA isolation***Total RNA was isolated from cell pellets or fresh frozen tissue samples (app. 20 mg) using RNeasy Mini Kit (Qiagen) according to the manufacturer´s instructions. The quality of the total RNA (>200 bases) was accessed using a 2100 Bioanalyzer (Agilent Technologies). All samples showed good integrity of the 18S and 28S ribosomal bands (RNA Integrity Number (RIN) > 5). Since the RNeasy spin columns specifically purifies RNA molecules longer than 200 bases the flow-through fraction contains small RNAs (<200 bases) including miRNAs. The small RNAs were recovered from the flow-through fraction using RNeasy Micro Kit together with the RNeasy MinElute spin columns (Qiagen). To allow binding of small RNAs to the RNeasy MinElute column the binding conditions were adjusted by adding 0.65 volume 100% EtOH to the flow-through fractions (instead of 1 volume 70% EtOH). The RNA was eluted from the columns with 14 µl of RNase free water.

***miRNA profiling***

The miRNA expression profiling was performed using the stem loop RT-qPCR based TaqMan Human MicroRNA Array Set v2.0 (Applied Biosystems) [2]. The Array Set consists of two arrays (A and B) containing a total of 667 unique miRNA assays representing mature human miRNAs present in the Sanger miRBase v10. In addition, endogenous control assays and a negative control assay are present on each array. The purified small RNA was reverse transcribed, pre-amplified and loaded onto the TaqMan A or B arrays as indicated by the manufacturer. Raw Cq values were calculated using the RQ manager software v1.2.1 (Applied Biosystems) with automatic baseline settings and a threshold of 0.2. The NormFinder algorithm was used to identify reference genes among the miRNAs expressed in the colorectal tissue samples [3]. The miR-340 was identified as an appropriate reference gene among the miRNAs present on the A array and miR-151-3p as reference gene among the miRNA present on the B array (data not shown). To prepare the raw Cq values for analysis they were processed using the following formula 2^-(Cqtarget-Cqref)^ and subsequently log2 transformed.

***miRNA and mRNA RT-qPCR***

Single tube TaqMan microRNA or mRNA assays (Applied Biosystems) were used to quantify individual mature miRNAs or mRNAs. The Applied Biosystems TaqMan Assay ID’s and the primer used for detection of the mRNA reference gene ubiquitin C (UBC) are listed in Supplementary Table S1. For miRNA quantification a pre-amplification step was included according to the manufacturer´s instructions. Quantification was performed using the standard curve method. The mRNA expression data were normalized using UBC as reference gene. The miRNA expression data were normalized using either miR-340 or miR-151-3p for fresh frozen tissue sample (cohort 1) or RNU44 for tissue samples in Solution D (cohort 2) and laser capture microdissected tissue samples. RNU6B or miR-340 was used for normalization of data from cell line pellets. TaqMan Assay IDs and the primers used for detection of UBC are listed in Supplementary Table S1.

***Validation of the knock-down of potential miR-375 targets using siRNA***

HCT116 cells (5 x 10^3^) were seeded in 96 well plates and reverse-transfected with 20 nM *Silencer* Select siRNAs (Applied Biosystems) designed to knock down HELLS, NOLC1, 20 nM of *Silence* Select negative control # siRNA (Applied Biosystems) or 20 nM custom siRNAs designed to knock down YAP1 (GenePharma, Shanghai, China). To verify the knock-down of HELLS, NOLC1 and YAP1 mRNAs, total RNA was isolated from siRNA transfected cells followed by RT-qPCR as described above. The Applied Biosystems *Silencer Select* siRNA ID’s and the sequences of the YAP1 siRNAs from GenePharma are listed in Supplementary Table S2.

***MTT assay***

# Cell viability/proliferation was measured using 3-[4,5-dimethylthiazol-2-yl]-2.5-diphenyltetrazolium bromide (MTT) assay (Roche Applied Science). DLD1 TR7, HCT116 (5 x 10^3^), LS174T TR4, HT29 cells (10 x10^3^) and SW480 (6 x 10^3^) were seeded in 96-well plates and reverse-transfected with 20 nM of precursor-miRNA (pre-miRNA), or 20 nM of Pre-miR™ miRNA Precursor Molecules-Negative Control #1 (scr-pre-miR)(Applied Biosystems) or 20 nM of *Silencer* Select siRNA (Applied Biosystems), 20 nM of *Silence* Select negative control # 1 siRNA (scr-siRNA) (Applied biosystem) or 20 nM custom siRNAs (YAP1) (GenePharma) using Lipofectamine 2000 (Invitrogen) for 72 hours. Subsequently, the cells were incubated with MTT substrate at 37^o^C in an atmosphere of 5% CO_2_ for 2 hours followed by lysis in 100 µl solubilisation buffer (20% [w/v] sodium dodecyl sulphate and 18.5% [v/v] formaldehyde) in the dark over night at room temperature. The absorbance in each well was measured at 540 nm and 690 nm on a multiplate reader; Multiscan MCC/340 (ThermoFisher Scientific)*.* The Applied Biosystems pre-miR, *Silencer* Select siRNA ID’s and the sequences of the YAP1 siRNAs from GenePharma are listed in Supplementary Table S2.

***Lactate dehydrogenase (LDH) assay***

Cellular death was measured using the lactate dehydrogenase (LDH) assay. DLD1 TR7, HCT116 (5 x 10^3^), LS174T TR4, HT29 cells (10 x10^3^) and SW480 (6 x 10^3^) were seeded in 96-well plates and reverse-transfected with 20 nM pre-miR-375 or 20 nM of scr-pre-miR (Applied Biosystems) or 20 nM of *Silence* Select siRNA (Applied Biosystems), 20 nM of scr-siRNA (Applied biosystem) or 20 nM custom siRNAs (YAP1) (GenePharma) for 48 hours using Lipofectamine 2000 (Invitrogen). Subsequently, the LDH activity was measured using the Cytotoxicity Detection Kit^PLUS^(LDH)(Roche Applied Science). The absorbance in each well was measured at 492 nm and 690 nm on a multiplate reader; Multiscan MCC/340 (ThermoFisher Scientific). The Applied Biosystems pre-miR, *Silencer* Select siRNA ID’s and the sequences of the YAP1 siRNAs from GenePharma are listed in Supplementary Table S2.

***Caspase 3/7 activity assay***

Apoptotic death was measured mainly as described previously using a Caspase3/7 activity assay [4]. DLD1 TR7, HCT116 (30 x 10^3^), LS174T TR4, HT29 cells (60 x10^3^) and SW480 (36 x 10^3^) were seeded in 24-well plates and reverse transfected with 20 nM pre miR-375 or 20 nM of scr-pre-miR (Applied Biosystems) or 20 nM of *Silencer* Select siRNA (Applied Biosystems), 20 nM of scr-siRNA (Applied Biosystems) or 20 nM custom siRNAs (YAP1) (GenePharma) for 48 hours using Lipofectamine 2000 (Invitrogen). The Applied Biosystems pre-miR**,** *Silencer* Select siRNA ID’s and the sequences of the YAP1 siRNAs from GenePharma are listed in Supplementary Table S2. Initially, subconfluent cells were lysed in caspase lysis buffer for 20 min on ice. Subsequently, one volume of 50 μM of Ac-DEVD-AFC substrate (Biomol, Plymouth Meeting, PA, USA) in Caspase reaction buffer was added to one volume of lysed cells in a black half area 96-well plate. The rate of substrate hydrolysis as measured by the liberation of AFC (excitation, 400 nm; emission 489 nm) over 20 min at 30°C was analyzed using a multiplate reader; Multiscan MCC/340 (ThermoFisher Scientific). The results were normalized to cellular death in general in the cell lysates as determine by an LDH assay.

***Laser Capture Microdissection***

Laser capture microdissection (LCM) was performed on cryosections from paired cancer and adjacent normal colon mucosa biopsies. Briefly, the sections were fixed in 95% EtOH for 120 sec, followed by 15 sec of staining in Arcturus Histogene Staining Solution (Applied Biosystems), dehydration in 95% EtOH (30 sec) and 100% EtOH (120 sec) before a final treatment in xylene for 120 sec. After drying of the slides epithelial cells were captured on individual caps using the Veritas 704 Microdissection Instrument (Applied Biosystems). Cells of interest were selected and captured using ultraviolet laser cutting according to the instructions given by the manufacturer. Subsequently, captured cells were incubated with RLT buffer (Qiagen) for 20 minutes at room temperature in the presence of 30 mM β-mercaptoethanol. Hematoxilin and eosin stained frozen sections were prepared for each tissue to reveal the area of interest for LCM. The miRNA extraction and the RT-qPCR were performed as described previously. In all, LCM was performed on 6 tissue sections (normal (n=3) and adenocarcinomas (n=3).

***mRNA profiling of HCT116 cells over-expressing miR-375 and clinical samples***

HCT116 cells (3 x 10^5^) were reverse-transfected with pre-miR-375 or Scr and harvested after 48 hours followed by isolation of total RNA (>200 bases) from cell pellets as described above. The experiment was performed in triplicates. Total RNA (100 ng) was labeled using the Ambion WT Expression Kit (Ambion) according to the manufacturer’s instructions. Washing, hybridization and scanning of the GeneChip Human Gene 1.0 ST Arrays (Affymetrix, Santa Clara, CA, USA) were performed according to the manufacturer’s protocol. To identify mRNAs that were negatively correlated to miR-375 in clinical samples, mRNA expression was profiled in 11 normal mucosa and 12 adenocarcinomas from cohort 1). Labeling of the total RNA (100 ng) and hybridization to the GeneChip Human Exon 1.0 ST Arrays (Affymetrix,Santa Clara, CA, USA) were performed as previously describe [5]. RMA16 quantile normalization was performed in the GeneSpring GX 12.1 software (Agilent Technologies, Santa Clara, CA, USA) as described previously [6].

***Identification of potential miR-375 targets based on mRNA profiling and in silico target prediction***

Only mRNAs identified as expressed above background (i.e. probe sets targeting only a single gene and with a median log intensity higher than 7) in our mRNA-profiles of miR-375 transfected HCT116 cells were included in the target analysis. The location and number of miR-375 seed sequences (i.e. complementary to position 2-8 of the miRNA) within the full length mRNA sequences was mapped using sequence data retrieved from the TargetScan v5.2 database (<http://www.targetscan.org/>) and Ensembl 62 database (<http://www.ensembl.org/index.html>).

Among the most differentially expressed genes in HCT116 cells over-expression miR-375, we identified 224 genes which were down-regulated (FC log2 < -0.5, p ≤ 0.05) and predicted to be direct miR-375 targets (predicted target by TargetScan and/or a 3’UTR containing one or more 7mer-m8, 7mer-A1 or 8mer seed matches). To identify biologically relevant miR-375 targets potentially playing a role in CRC, we analyzed the correlation between the expression of the 224 putative miR-375 targets and miR-375 in clinical CRC samples. These analyses were performed in a subset of the CRC samples used for miRNA profiling (normal colon mucosa n=10 and adenocarcinoma n=11). Using a Pearson correlation of -0.6 as cutoff we identified 18 putative miR-375 targets that were negatively correlated to miR-375 in clinical CRC samples.

***Protein extraction and Western blotting***

Protein extraction and western blotting analysis were performed according to standard procedures. Briefly, cells were lysed in 1 x Cell Lysis Buffer (Cell Signaling Technology, Beverly, MA, USA). Protein concentrations were estimated with Bio-Rad Protein assay (Bradford) (BioRad, Hercules, CA, USA). Approximately 20 μg of protein was loaded onto NuPAGE precast 4-12% gels (Invitrogen, Carlsbad, CA, USA). Gel electrophoresis and protein transfer were performed using XCell SureLock Mini-Cell with Blot Module™ (Invitrogen). Membranes were blocked (0.1% PBS and 5% Tween) and incubated for at least 16 hours at room temperature with mouse anti-HELLS (Santa Cruz (sc46665), Dallas TX, USA; dilution 1:500), mouse anti-NOLC1 (Santa Cruz (sc374033), Dallas TX, USA; dilution 1:1000) and rabbit anti-YAP1 (abcam (ab52771), Cambridge, UK; dilution 1:25.000). Following incubation with Goat anti-mouse or Swine anti-rabbit horseradish peroxidase HRP-conjugated secondary antibody (Dako, Glostrup, DK; dilution 1:3000) proteins were detected using ECL prime Western Blotting Reagent (GE Healthcare, Chalfont St. Giles, UK) and ChemiDoc-It Imaging System (UVP, Upland, CA, USA). The β-actin was visualized using HRP-conjugated mouse anti-β-actin (Abcam; dilution 1:3.000) or HRP-conjugated rabbit anti-β-actin (abcam; dilution 1:3.000).

***Construction of plasmids for the Luciferase reporter assay***

Selected fragments of the 3’UTRs of HELLS (NM_018063) and NOLC1 (NM_004741), containing the putative miR-375 binding sites were amplified from human genomic DNA. The primers introduced an *Xho*1 and a *Not*1 restriction enzyme digestion site at the 5´-and 3´-end of the amplicons, respectively, allowing insertion of the putative miRNA targets sites downstream of the *Renilla* luciferase gene in the siCHECK-2 vector (Promega). This vector also harbours a *Firefly* Luciferase gene, which is used for normalization. Selected constructs were mutated in the putative miRNA binding region using QuickChange Lightning Site Directed Mutagenesis Kit (Agilent Technologies). More specifically, the seed matches were replaced in a combinatorial manner with an *EcoRI* enzyme restriction site. This completely abolished the putative miR-375 binding sites and allowed for easy identification of mutated constructs using *EcoRI* enzyme digestion analysis. All constructs were verified by sequencing using the BigDye Terminator v1.1 Cycle Sequencing kit (Applied Biosystems). The primers used for cloning and site directed mutagenesis are described in Supplementary Table S3.

***Luciferase reporter assay***

A total of 20 x 10^3^ Human Embryonic Kidney T293 (HEKT293) cells were seeded in 96-well plates in 100 µl D-MEM without penicillin/streptomycin 24 hours prior to transfection. Cells were co-transfected with 50 nM (final concentration) pre-miR-375 or scr-pre-miR (Applied Biosystems) and 80 ng of the siCHECK-2 vectors using Lipofectamine 2000 (Invitrogen). Each treatment was performed in triplicates and repeated in three independent experiments. Twenty-four hours after transfection the cells were collected and analysed using Dual Glo Luciferase Assay System (Promega) with some modifications as described previously [7]. Briefly, cells were lysed on ice for 20 min by adding 60 µl of lysis buffer (0.5% [v/v] Triton X-100, 10% [v/v] glycerol, 20 mM Tris HCl pH 7.8, 1 mM DTT and a Protease Inhibitor Cocktail Tablet (Roche Applied Science)). Subsequently, 20 µl of the lysate were used to determine Luciferase activity by successive addition of 20 µl of *Firefly* and *Renilla* substrate, respectively. Luminescence was detected with a multiplate reader; Multiscan MCC/340 (ThermoFisher Scientific). The *Renilla* Luciferase activity was normalized to the *Firefly* Luciferase activity for each transfected well, to correct for differences in transfection and harvest efficiencies and normalized to Scr. Negative controls were; co-transfection of an empty vector with pre-miR-375 or scr-pre-miR (data not shown).

***Generation and characterization of stable HCT116 with inducible miR-375 expression***

Initially, *MIR-375* was cloned into the 3’ UTR region of the *turbo red fluorescence protein gene* (tRFP) of the pSBInducer10 vector (*MIR375*_pSBInducer10) using Expand High Fidelity DNA Polymerase (Roche Applied Science) and a pool of genomic DNA isolated from human blood as template. The primers amplified the miR-375 precursor (*MIR375 (*Gene ID: 494324)) and 170 bases up/down stream of *MIR375* (primer sequences are given in Supplementary Table S1). Mlu1 and Not1 restriction enzyme sites were included in the 5’-end of the sense and antisense primer, respectively. The pSBInducer10 vector was constructed by replacing lentiviral elements in the pINDUCER vector [8] with SleepingBeauty inverted terminal repeats. The PCR product containing *MIR375* and the pSBInducer10 vector were digested with Mlu1 and Not1 followed by ligation and transformation of the ligation product into One shot TOP10 chemically competent *E. coli* (Invitrogen). The cloned sequences were verified using Sanger Sequencing. Stable HCT116 cells with inducible expression of miR-375 were generated as follows. Firstly, 1.5 x 10^6^ HCT116 cells were seed and transfected with *MIR375*_pSBInducer10 or *Scr*_pSBInducer10 and pCMV-SB100XCO (vector with transposase) after 24 hours using Opti-MEM (Life Technolgies). Forty-eight hours post-transfection puromycin (final concentration 1 µg/mL) (Sigma) was added to select for stably transfected cells. The puromycin selection was carried out for 5 days. Subsequently, the cells were treated with 50 µg/ml doxycycline (dox) (Sigma) for 48 hours leading to transcriptional activation of the tRFP-*MIR375* cassette. The tRFP fluorescence marker was used as a surrogate to sort for cell populations expressing high levels of miR-375 after induction of dox. Briefly, the cells with the highest tRFP level (100-1000 times above the background level in untreated cells) (HCT116_miR-375H and HCT116_ScrH) were isolated by fluorescence-activated cell sorting (FACS) using a 4-laser FACSAriaIII (BD Biosciences, San Jose, CA) and used for all subsequent analyses. Dox dependent expression of mature miR-375 in the HCT116_miR-375H cells was analyzed using RT-qPCR as describe earlier. The HCT116_miR-375H cells were phenotypically characterized using xCELLigence (Roche Applied Science), Caspase 3/7 assays and YAP1 Western blotting. The xCELLigence system was used for real-time monitoring of cell proliferation [9]. Briefly, HCT116-miR-375H cells (7 x 10^3^) were seeded in E-plates (Roche Applied Science) according to the manufacturer’s instructions. All experiments were performed with three biological replicates. Dox was added at time 0. The cells were monitored for 0-96 hours. Following the real-time monitoring, the slope (rate of changes in cell index) was calculated from time 60-80 hours (i.e. when changes in cell viability were apparent). Western blotting and the Caspase 3/7 assay were carried out as described previously except that Dox was added at time 0.

***References Supplementary Material***

1. Rantala JK, Makela R, Aaltola AR, Laasola P, Mpindi JP et al. (2011) A cell spot microarray method for production of high density siRNA transfection microarrays. BMC Genomics 12: 162.

2. Chen C, Ridzon DA, Broomer AJ, Zhou Z, Lee DH et al. (2005) Real-time quantification of microRNAs by stem-loop RT-PCR. Nucleic Acids Res 33: e179.

3. Andersen CL, Jensen JL, Orntoft TF (2004) Normalization of real-time quantitative reverse transcription-PCR data: a model-based variance estimation approach to identify genes suited for normalization, applied to bladder and colon cancer data sets. Cancer Res 64: 5245-5250.

4. Ostenfeld MS, Fehrenbacher N, Hoyer-Hansen M, Thomsen C, Farkas T et al. (2005) Effective tumor cell death by sigma-2 receptor ligand siramesine involves lysosomal leakage and oxidative stress. Cancer Res 65: 8975-8983.

5. Thorsen K, Mansilla F, Schepeler T, Oster B, Rasmussen MH et al. (2011) Alternative splicing of SLC39A14 in colorectal cancer is regulated by the Wnt pathway. Mol Cell Proteomics 10: M110.

6. Oster B, Thorsen K, Lamy P, Wojdacz TK, Hansen LL et al. (2011) Identification and validation of highly frequent CpG island hypermethylation in colorectal adenomas and carcinomas. Int J Cancer 129: 2855-2866.

7. Zhang S, Nohturfft A (2008) Studying Membrane Biogenesis with a Luciferase-Based Reporter Gene Assay. J Vis Exp 19: e920.

8. Meerbrey KL, Hu G, Kessler JD, Roarty K, Li MZ et al. (2011) The pINDUCER lentiviral toolkit for inducible RNA interference in vitro and in vivo. Proc Natl Acad Sci U S A 108: 3665-3670.

9. Atienza JM, Yu N, Kirstein SL, Xi B, Wang X et al. (2006) Dynamic and label-free cell-based assays using the real-time cell electronic sensing system. Assay Drug Dev Technol 4: 597-607.
