# Supplementary material for: Functional Screening Identifies miRNAs Influencing Apoptosis and Proliferation in Colorectal Cancer
Source: PLoS One. 2014 Jun 3;9(6):e96767. doi: 10.1371/journal.pone.0096767 (PMC4043686; doi:10.1371/journal.pone.0096767)
Supplement: File S1 — Tables S1–S3, S6 and S7. (DOCX) [file pone.0096767.s010.docx]

**Supplementary tables**

**Table S1. Applied Biosystems TaqMan Assay Id’s and primer sequences**

| **Assay/primer name** | **Assay Id/primer sequences** |
| --- | --- |
| **mRNA** |  |
| HELLS | Hs00934790_m1 |
| NOLC1 | Hs01102319_g1 |
| YAP1 | Hs00902712_g1 |
| BCL2L1 | Hs00236329_m1 |
| BIRC5 (Survivin) | Hs04194392_s1 |
|  |  |
| **miRNAs** |  |
| hsa-miR-375 | 000564 |
| hsa-miR-340 | 002258 |
| hsa-miR-151-3p | 002254 |
| RNU44 | 001094 |
| RNU6B | 001093 |
|  |  |
| **UBC Primers** |  |
| UBC-forward | 5’-GATTTGGGTCGCGGTTCTT-3’ |
| UBC-reverse | 5’-TGCCTTGACATTCTCGATGGT-3’ |
|  |  |
| **ChIP primers** |  |
| 1242-forward | 5’-CTTCACCCGAGAAATTCAGC-3’ |
| 1242-reverse | 5’-CGTGTGAACACGTCGCTAAG-3’ |
| 1243_1-forward | 5’-GAATCCAGAGCTGCTTCCAG-3’ |
| 1243_1-reverse | 5’-AGTGCATTGGAGATGGGAGA-3’ |
| 1243_2 forward | 5’-GCCCATGGGAATTTCTTTTT-3’ |
| 1243_2 reverse | 5’-AAGCAGCTCTGGATTCCTCA-3’ |
|  |  |
| **Cloning of miR-375 genomic region** |  |
| miR-375_Mlu1 forward | 5’-cagcacgcgtAGAAGCTGCGGGCAGGGCAAGGC-3’ |
| miR-375_Not1reverse | 5’-ctcggcggccgcTCAGCCGCAGATGCGTTCAGGTG-3’ |

The Mlu1 and Not1 restriction enzyme sites are underlined.

**Table S2 MiRNA mimics and siRNA from Applied Biosystems and GenePharma**

| **Assay Name** | **Assay Id/**  **Part Number** | **Sequence** |
| --- | --- | --- |
| **siRNA** |  |  |
| HELLS siRNA_1 | S6506 | - |
| HELLS siRNA_2 | S6505 | - |
| NOLC1 siRNA_1 | S17634 | - |
| NOLC1 siRNA_2 | S17633 | - |
| Silencer Select Negative Control #1 siRNA | 4390843 | - |
| YAP1 siRNA_1 | - | 5´-CGAUUAUCUGCUCUCUCUUUU-3’ |
|  |  | 5´- AAGAGAGAGCAGAUAAUCGUU-3’ |
| YAP1 siRNA_2 | - | 5’-GCUAUAUAAACCAUAAGAAUU-3’ |
|  |  | 5’-UUCUUAUGGUUUAUAUAGCUU-3’ |
| Negative control | - | 5’-UUCUCCGAACGUGUSACGUTT-3’ |
|  |  | 5’-ACGUGACACGUUCGGAGAATT-3’ |
| **miRNA mimics** |  |  |
| hsa-miR-375 | PM10327 | - |
| hsa-miR-9 | PM10022 | - |
| hsa-miR-138 | PM11727 | - |
| hsa-miR-23b | PM10711 | - |
| hsa-miR-150 | PM10070 | - |
| hsa-miR-145 | PM11480 | - |
| Pre-miR miRNA Precursor Molecules- Negative Control #1 | AM17110 | **-** |

**Table S3. Primers used for 3´-UTR cloning and mutagenesis**

| **Primer name** | **Primer sequence** |
| --- | --- |
| HELLS-Xho-1 FW | 5’-ATATCTCGAGAGTGGAGCTCAAGAATAGC-3’ |
| HELLS-Not-1 RV | 5’-ATAAGCGGCCGCGAGCATAATCCCAATCTCTCCC-3’ |
| HELLS_mut1 FW | 5’-AAATAGATGGTAATTTTCTGAGCCTTACCAAGAA**TTC**AGAAGTATCCATATTAAGTTTAGATTTTCAG-3’ |
| HELLS_mut1 RV | 5’-CTGAAAATCTAAACTTAATATGGATACTTCT**GAA**TTCTTGGTAAGGCTCAGAAAATTACCATCTATTT-3’ |
| NOLC1-Xho-1 FW | 5’-ATATCTCGAGCCTGAGGCCATCTTCGGTGAAG-3’ |
| NOLC1-Not-1 RV | 5’-ATATGCGGCCGCCCATGCAGACTGAAATATCTGTGTCAGC-3’ |
| NOLC1_mut1 FW | 5’-GACTGCATCTTCTCGTTTTTTACAGTATAGAGAA**TTC**AGTGACATGAGTTTGAAAAATACATATCACTTGG-3’ |
| NOLC1_mut1 RV | 5’-CCAAGTGATATGTATTTTTCAAACTCATGTCACT**GAA**TTCTCTATACTGTAAAAAACGAGAAGATGCAGTC-3’ |
| NOLC1_mut2 FW | 5’-TGATAAACTGGAGTGGGTTTGGGAGAA**TTC**ACTAATAGGATTATTGTGTCTC-3’ |
| NOLC1_mut2 RV | 5’-GAGACACAATAATCCTATTAGT**GAA**TTCTCCCAAACCCACTCCAGTTTATCAG-3’ |

The Not1 and Xho1 sites in the primers are underlined.

Mutations introduced by the mutagenesis primers are underlined and in bold.

The mutagenesis primers were HPLC purified.

**Table S6. Clinically dys-regulated miRNAs inducing phenotypic changes in CRC cell lines**

| **miR ID** | **Number of cell lines ^§^** | **Known phenotypic effect of ectopic expression** | **Dys-regulation in CRC** | | **Dys-regulation other cancers** |
| --- | --- | --- | --- | --- | --- |
| **Increased cPARP cleavage** |  |  |  |  | |
| hsa-miR-517a^Δ^ | 2 | Induces apoptosis^[1]^  Increases proliferation^[2]^ | Up-reg | | Up-reg: hepatocellular carcinoma^[2]^ |
| hsa-miR-224 | 4 | Induces apoptosis and increases proliferation^[3]^ | Up-reg | | Up-reg: hepatocellular carcinoma, prostate and renal cancer^[3–5]^  Down –reg: oral cancer^[6]^ |
| hsa-miR-29b | 1 | Induces apoptosis and reduces proliferation^[7,8]^  Reduces apoptosis^[9]^ | Up-reg | | Up-reg: breast cancer^[10,11]^  Down-reg: ovarian cancer and leukemia^[12,13]^ |
| hsa-miR-150^Δ^ | 2 | Induces apoptosis and reduces proliferation^[14,15]^  Increases proliferation^[16]^ | Down-reg | | Down-reg: lymphoma^[14,15]^  Up-reg:gastric cancer^[16]^ |
| hsa-miR-139-5p | 1 | - | Down-reg | | Down-reg: bladder and endometrial cancer^[17,18]^ |
| hsa-miR-375^Δ^ | 1 | Induces apoptosis and reduces proliferation^[19–26]^ | Down-reg | | Down-reg: liver, gastic cervical, head and neck cancer and melanoma^[19–23]^ |
| **Decreased Ki67** |  |  |  | |  |
| hsa-miR-9^Δ^ | 4 | Reduces proliferation^[27–29]^ | Down-reg | | Down-reg: ovarian and gastric cancer^[27,30]^  Up-reg: brain tumors^[31]^ |
| hsa-miR-23b^Δ^ | 2 | Reduces proliferation^[32]^ | Down-reg | | Down-regulated: liver cancer^[32]^  Up-regulated: bladder, gastric and oral cancer^[33–35]^ |
| hsa-miR-424^Δ^ | 1 | - | Up-reg | | Down-reg: leukemia^[36]^  Up-reg: tongue cancer^[37]^ |
| hsa-miR-138^Δ^ | 1 | Induces apoptosis and reduces proliferation^[38,39]^ | Down-reg | | Up-reg: thyroid cancer^[40]^  Down-reg: Tongue, head and neck cancer^[41,42]^ |

^Δ^pre-miRs selected for further *in vitro* analysis

^§^Number of cell lines demonstrating a z-score ≥1.5 (c-PARP cleavage) or ≤ -1.5 (decreased Ki67)

**Table S7. Enriched list of miR-375 target candidates**

| **Gene symbol** | **FC (log2)** | **p-value**  **(corrected)** | **Predicted by TargetScan** | **3'UTR**  **seed**  **match** | **Pearson (clinical samples)** |
| --- | --- | --- | --- | --- | --- |
| PHLDA1 | -0.56 | 0.023 | + | 7mer-A1 | -0.76 |
| MKI67† | -0.61 | 0.029 | - | 7mer-A1 | -0.73 |
| CTPS | -0.93 | 0.013 | - | 7mer-A1 | -0.72 |
| KIAA1430 | -0.6 | 0.034 | - | 7mer-A1 | -0.7 |
| WWC2 | -1.05 | 0.012 | + | 7mer-A1 (2) | -0.69 |
| APBB2† | -0.91 | 0.027 | - | 8mer | -0.68 |
| MLLT1 | -0.93 | 0.01 | + | 7mer-A1 | -0.67 |
| STX6 | -0.67 | 0.016 | - | 7mer-A1 | -0.67 |
| **NOLC1†** | **-0.6** | **0.018** | **-** | **7mer-A1 (2)** | **-0.67** |
| FAM105B | -0.72 | 0.042 | - | 7mer-A1 (2) | -0.66 |
| C1orf107 | -0.58 | 0.023 | - | 7mer-A1 | -0.66 |
| XPR1 | -0.7 | 0.028 | - | 7mer-A1 (2) | -0.65 |
| HCFC1† | -0.61 | 0.016 | - | 7mer-A1 | -0.65 |
| **HELLS†** | **-0.72** | **0.01** | **-** | **7mer-A1** | **-0.64** |
| ENOPH1 | -0.7 | 0.011 | - | 7mer-A1 | -0.62 |
| ZNF695 | -1.19 | 0.021 | - | 7mer-m8 | -0.6 |
| SKI† | -0.73 | 0.047 | + | 7mer-A1 | -0.6 |
| ^Δ^YAP1 | -0.57 | 0.039 | - | 7mer-A1 (2) | -0.6 |

Selection criteria:

1) Significantly down-regulated upon miR-375 over expression (FC _(log2)_ > -0.5, p < 0.05) and containing one or more 7mer-m8, 7mer-A1 or 8mer seed matches* in the 3’UTR (224 genes)

and

2) Significantly up-regulated (FC (log2) > -0.5, p < 0.05) and negatively correlated with miR-375 in clinical samples (Pearson correlation ≤ -0.60) (18 genes listed). The miR-375 target candidates selected for further analysis are in bold.

^Δ^YAP1 has been identified as a direct miR-375 target in liver cancer

*Predicted target by TargetScan and/or 3’UTR containing one or more 7mer-m8, 7mer-A1 or 8mer seed matches

†Predicted to be involved in pathways related to cell cycle regulation using Ingenuity Pathway Analysis (IPA)

**References Supplementry Tables**

1. Yoshitomi T, Kawakami K, Enokida H, Chiyomaru T, Kagara I et al. (2011) Restoration of miR-517a expression induces cell apoptosis in bladder cancer cell lines. Oncol Rep 25: 1661-1668.

2. Toffanin S, Hoshida Y, Lachenmayer A, Villanueva A, Cabellos L et al. (2011) MicroRNA-based classification of hepatocellular carcinoma and oncogenic role of miR-517a. Gastroenterology 140: 1618-1628.

3. Wang Y, Lee AT, Ma JZ, Wang J, Ren J et al. (2008) Profiling microRNA expression in hepatocellular carcinoma reveals microRNA-224 up-regulation and apoptosis inhibitor-5 as a microRNA-224-specific target. J Biol Chem 283: 13205-13215.

4. Boguslawska J, Wojcicka A, Piekielko-Witkowska A, Master A, Nauman A (2011) MiR-224 Targets the 3'UTR of Type 1 5'-Iodothyronine Deiodinase Possibly Contributing to Tissue Hypothyroidism in Renal Cancer. PLoS One 6: e24541.

5. Prueitt RL, Yi M, Hudson RS, Wallace TA, Howe TM et al. (2008) Expression of microRNAs and protein-coding genes associated with perineural invasion in prostate cancer. Prostate 68: 1152-1164.

6. Scapoli L, Palmieri A, Lo ML, Pezzetti F, Rubini C et al. (2010) MicroRNA expression profiling of oral carcinoma identifies new markers of tumor progression. Int J Immunopathol Pharmacol 23: 1229-1234.

7. Garzon R, Heaphy CE, Havelange V, Fabbri M, Volinia S et al. (2009) MicroRNA 29b functions in acute myeloid leukemia. Blood 114: 5331-5341.

8. Zhang YK, Wang H, Leng Y, Li ZL, Yang YF et al. (2011) Overexpression of microRNA-29b induces apoptosis of multiple myeloma cells through down regulating Mcl-1. Biochem Biophys Res Commun 414: 233-239.

9. Wang C, Bian Z, Wei D, Zhang JG (2011) miR-29b regulates migration of human breast cancer cells. Mol Cell Biochem 352: 197-207.

10. Yan LX, Huang XF, Shao Q, Huang MY, Deng L et al. (2008) MicroRNA miR-21 overexpression in human breast cancer is associated with advanced clinical stage, lymph node metastasis and patient poor prognosis. RNA 14: 2348-2360.

11. Cortez MA, Nicoloso MS, Shimizu M, Rossi S, Gopisetty G et al. (2010) miR-29b and miR-125a regulate podoplanin and suppress invasion in glioblastoma. Genes Chromosomes Cancer 49: 981-990.

12. Flavin R, Smyth P, Barrett C, Russell S, Wen H et al. (2009) miR-29b expression is associated with disease-free survival in patients with ovarian serous carcinoma. Int J Gynecol Cancer 19: 641-647.

13. Garzon R, Volinia S, Liu CG, Fernandez-Cymering C, Palumbo T et al. (2008) MicroRNA signatures associated with cytogenetics and prognosis in acute myeloid leukemia. Blood 111: 3183-3189.

14. Ghisi M, Corradin A, Basso K, Frasson C, Serafin V et al. (2011) Modulation of microRNA expression in human T-cell development: targeting of NOTCH3 by miR-150. Blood 117: 7053-7062.

15. Watanabe A, Tagawa H, Yamashita J, Teshima K, Nara M et al. (2011) The role of microRNA-150 as a tumor suppressor in malignant lymphoma. Leukemia 25: 1324-1334.

16. Wu Q, Jin H, Yang Z, Luo G, Lu Y et al. (2010) MiR-150 promotes gastric cancer proliferation by negatively regulating the pro-apoptotic gene EGR2. Biochem Biophys Res Commun 392: 340-345.

17. Yoshino H, Chiyomaru T, Enokida H, Kawakami K, Tatarano S et al. (2011) The tumour-suppressive function of miR-1 and miR-133a targeting TAGLN2 in bladder cancer. Br J Cancer 104: 808-818.

18. Hiroki E, Akahira J, Suzuki F, Nagase S, Ito K et al. (2010) Changes in microRNA expression levels correlate with clinicopathological features and prognoses in endometrial serous adenocarcinomas. Cancer Sci 101: 241-249.

19. Liu AM, Poon RT, Luk JM (2010) MicroRNA-375 targets Hippo-signaling effector YAP in liver cancer and inhibits tumor properties. Biochem Biophys Res Commun 394: 623-627.

20. Ding L, Xu Y, Zhang W, Deng Y, Si M et al. (2010) MiR-375 frequently downregulated in gastric cancer inhibits cell proliferation by targeting JAK2. Cell Res 20: 784-793.

21. Wang F, Li Y, Zhou J, Xu J, Peng C et al. (2011) miR-375 is down-regulated in squamous cervical cancer and inhibits cell migration and invasion via targeting transcription factor SP1. Am J Pathol 179: 2580-2588.

22. Hui AB, Lenarduzzi M, Krushel T, Waldron L, Pintilie M et al. (2010) Comprehensive MicroRNA profiling for head and neck squamous cell carcinomas. Clin Cancer Res 16: 1129-1139.

23. Mazar J, DeBlasio D, Govindarajan SS, Zhang S, Perera RJ (2011) Epigenetic regulation of microRNA-375 and its role in melanoma development in humans. FEBS Lett 585: 2467-2476.

24. Tsukamoto Y, Nakada C, Noguchi T, Tanigawa M, Nguyen LT et al. (2010) MicroRNA-375 is downregulated in gastric carcinomas and regulates cell survival by targeting PDK1 and 14-3-3zeta. Cancer Res 70: 2339-2349.

25. Nohata N, Hanazawa T, Kikkawa N, Mutallip M, Sakurai D et al. (2011) Tumor suppressive microRNA-375 regulates oncogene AEG-1/MTDH in head and neck squamous cell carcinoma (HNSCC). J Hum Genet 56: 595-601.

26. He XX, Chang Y, Meng FY, Wang MY, Xie QH et al. (2011) MicroRNA-375 targets AEG-1 in hepatocellular carcinoma and suppresses liver cancer cell growth in vitro and in vivo. Oncogene 31: 3357-3369.

27. Guo LM, Pu Y, Han Z, Liu T, Li YX et al. (2009) MicroRNA-9 inhibits ovarian cancer cell growth through regulation of NF-kappaB1. FEBS J 276: 5537-5546.

28. Minor J, Wang X, Zhang F, Song J, Lu X et al. (2011) Methylation of microRNA-9 is a specific and sensitive biomarker for oral and oropharyngeal squamous cell carcinomas. Oral Oncol 48: 73-78.

29. Wan HY, Guo LM, Liu T, Liu M, Li X et al. (2010) Regulation of the transcription factor NF-kappaB1 by microRNA-9 in human gastric adenocarcinoma. Mol Cancer 9: 16.

30. Luo H, Zhang H, Zhang Z, Zhang X, Ning B et al. (2009) Down-regulated miR-9 and miR-433 in human gastric carcinoma. J Exp Clin Cancer Res 28: 82.

31. Nass D, Rosenwald S, Meiri E, Gilad S, Tabibian-Keissar H et al. (2009) MiR-92b and miR-9/9* are specifically expressed in brain primary tumors and can be used to differentiate primary from metastatic brain tumors. Brain Pathol 19: 375-383.

32. Salvi A, Sabelli C, Moncini S, Venturin M, Arici B et al. (2009) MicroRNA-23b mediates urokinase and c-met downmodulation and a decreased migration of human hepatocellular carcinoma cells. FEBS J 276: 2966-2982.

33. Gottardo F, Liu CG, Ferracin M, Calin GA, Fassan M et al. (2007) Micro-RNA profiling in kidney and bladder cancers. Urol Oncol 25: 387-392.

34. Li X, Zhang Y, Zhang H, Liu X, Gong T et al. (2011) miRNA-223 promotes gastric cancer invasion and metastasis by targeting tumor suppressor EPB41L3. Mol Cancer Res 9: 824-833.

35. Scapoli L, Palmieri A, Lo ML, Pezzetti F, Rubini C et al. (2010) MicroRNA expression profiling of oral carcinoma identifies new markers of tumor progression. Int J Immunopathol Pharmacol 23: 1229-1234.

36. Pallasch CP, Patz M, Park YJ, Hagist S, Eggle D et al. (2009) miRNA deregulation by epigenetic silencing disrupts suppression of the oncogene PLAG1 in chronic lymphocytic leukemia. Blood 114: 3255-3264.

37. Rentoft M, Fahlen J, Coates PJ, Laurell G, Sjostrom B et al. (2011) miRNA analysis of formalin-fixed squamous cell carcinomas of the tongue is affected by age of the samples. Int J Oncol 38: 61-69.

38. Liu X, Jiang L, Wang A, Yu J, Shi F et al. (2009) MicroRNA-138 suppresses invasion and promotes apoptosis in head and neck squamous cell carcinoma cell lines. Cancer Lett 286: 217-222.

39. Jiang L, Dai Y, Liu X, Wang C, Wang A et al. (2011) Identification and experimental validation of G protein alpha inhibiting activity polypeptide 2 (GNAI2) as a microRNA-138 target in tongue squamous cell carcinoma. Hum Genet 129: 189-197.

40. Vriens MR, Weng J, Suh I, Huynh N, Guerrero MA et al. (2011) MicroRNA expression profiling is a potential diagnostic tool for thyroid cancer. Cancer 118: 3426-3432.

41. Wong TS, Liu XB, Wong BY, Ng RW, Yuen AP et al. (2008) Mature miR-184 as Potential Oncogenic microRNA of Squamous Cell Carcinoma of Tongue. Clin Cancer Res 14: 2588-2592.

42. Liu X, Chen Z, Yu J, Xia J, Zhou X (2009) MicroRNA profiling and head and neck cancer. Comp Funct Genomics id: 837514.
