# Supplementary material for: Functional Screening Identifies miRNAs Influencing Apoptosis and Proliferation in Colorectal Cancer
Source: PLoS One. 2014 Jun 3;9(6):e96767. doi: 10.1371/journal.pone.0096767 (PMC4043686; doi:10.1371/journal.pone.0096767)
Supplement: File S2 — Tables S4 and S5. (DOCX) [file pone.0096767.s011.docx]

**Supplementary Table S4. Rank product Top-40 pre-miRs (increased cPARP cleavage)**

| **Pre-miR ID** |  | **Z-scores** | | | | | |  | **Ranking** | | | | | |  | **Dys-regulation clinical samples** |
| --- | --- | --- | --- | --- | --- | --- | --- | --- | --- | --- | --- | --- | --- | --- | --- | --- |
|  |  | **HCT116** | **HT29** | **SW480** | **DLD1 TR7** | **LS174T TR4** | **Caco2** |  | **HCT116** | **HT29** | **SW480** | **DLD1 TR7** | **LS174T TR4** | **Caco2** |  |  |
| hsa-miR-506 |  | 2.4 | -1.8 | 3.2 | -0.4 | 3.7 | -0.1 |  | 6 | 311 | 1 | 214 | 1 | 170 |  | UD |
| hsa-miR-126 |  | 0.2 | 1.5 | 0.8 | 3.4 | 0.4 | 0.6 |  | 140 | 22 | 33 | 1 | 67 | 83 |  | NC |
| hsa-miR-182 |  | -0.1 | -0.3 | 2.5 | 0.3 | 2.1 | 1.7 |  | 177 | 195 | 2 | 121 | 2 | 21 |  | Up (1.1) |
| hsa-miR-211 |  | 1.7 | 0.6 | 2.3 | -2.0 | 0.9 | 0.1 |  | 16 | 84 | 4 | 313 | 13 | 132 |  | UD |
| **hsa-miR-373*** |  | 3.7 | -0.2 | 0.2 | 3.2 | -0.4 | -0.4 |  | 2 | 187 | 124 | 2 | 248 | 209 |  | UD |
| hsa-miR-517a |  | 3.3 | 3.5 | -0.2 | 0.5 | -0.3 | 0.1 |  | 3 | 2 | 202 | 104 | 230 | 135 |  | Up (2.7)^Δ^ |
| hsa-miR-126* |  | 0.7 | 0.6 | 1.9 | 0.5 | 1.1 | 2.6 |  | 76 | 86 | 6 | 102 | 9 | 6 |  | NC |
| **hsa-let-7b** |  | 2.5 | 1.9 | 0.4 | 1.0 | -1.0 | 1.3 |  | 4 | 11 | 90 | 49 | 302 | 32 |  | NC |
| hsa-miR-224 |  | 1.9 | 2.4 | -1.0 | 1.7 | -0.7 | 1.5 |  | 12 | 5 | 290 | 16 | 286 | 29 |  | Up (2.1) |
| hsa-miR-106b |  | -0.6 | 1.1 | 1.6 | -0.9 | 1.5 | 1.5 |  | 237 | 44 | 8 | 264 | 4 | 27 |  | Up (0.9) |
| hsa-miR-29b |  | 0.6 | -0.2 | 0.8 | 2.1 | 0.5 | 0.5 |  | 86 | 184 | 42 | 4 | 50 | 93 |  | Up (1.5) |
| hsa-miR-101 |  | 1.9 | 0.4 | 0.1 | 1.7 | 0.1 | -0.7 |  | 11 | 109 | 147 | 10 | 128 | 238 |  | NC |
| hsa-miR-150 |  | 1.6 | -0.6 | 0.4 | 2.3 | -0.3 | -0.1 |  | 19 | 246 | 80 | 3 | 217 | 163 |  | Down (1.7) |
| **hsa-let-7i** |  | 1.7 | 1.7 | 0.7 | 0.0 | 0.0 | -0.3 |  | 15 | 15 | 45 | 167 | 159 | 189 |  | NA |
| hsa-miR-508 |  | 1.7 | 0.7 | 0.7 | 1.2 | 0.1 | -1.5 |  | 18 | 74 | 49 | 37 | 118 | 302 |  | UD |
| **hsa-miR-369-5p** |  | 0.5 | 0.9 | -0.3 | 0.6 | 2.1 | -0.9 |  | 95 | 52 | 215 | 91 | 3 | 260 |  | NC |
| **hsa-miR-452*** |  | 0.4 | 0.5 | 0.7 | 1.6 | 0.6 | 0.8 |  | 114 | 90 | 51 | 18 | 40 | 56 |  | UD |
| **hsa-miR-183** |  | 1.1 | -0.5 | 1.5 | 0.5 | 0.6 | 1.8 |  | 48 | 221 | 9 | 98 | 43 | 18 |  | Up (1.3) |
| hsa-miR-518a-2* |  | 1.8 | 1.4 | 0.4 | 1.0 | -0.5 | 0.9 |  | 13 | 30 | 88 | 50 | 254 | 50 |  | NA |
| hsa-miR-19a |  | 3.9 | 0.1 | 0.1 | 0.5 | -0.4 | 0.9 |  | 1 | 143 | 142 | 103 | 242 | 51 |  | Up (1.3) |
| **hsa-miR-185** |  | -0.7 | 0.9 | -1.7 | 1.3 | 1.4 | 1.1 |  | 240 | 51 | 313 | 27 | 5 | 41 |  | NC |
| hsa-miR-181d |  | 0.8 | -0.4 | 1.1 | 0.6 | 0.8 | 0.3 |  | 64 | 209 | 23 | 86 | 21 | 115 |  | NA |
| hsa-miR-18a* |  | 0.2 | 0.4 | 1.4 | -0.8 | 1.0 | 0.3 |  | 141 | 114 | 13 | 248 | 11 | 107 |  | Up (0.7) |
| hsa-miR-302d |  | 0.7 | 1.4 | -0.1 | 1.4 | 0.3 | -1.9 |  | 71 | 25 | 185 | 25 | 92 | 315 |  | UD |
| hsa-miR-153 |  | 1.0 | 2.7 | -0.6 | 0.9 | -1.4 | 0.6 |  | 50 | 3 | 266 | 62 | 311 | 86 |  | NC |
| hsa-miR-187 |  | 1.9 | 2.1 | 0.0 | -0.5 | -0.3 | -0.2 |  | 10 | 10 | 160 | 224 | 218 | 179 |  | UD |
| hsa-miR-139-5p |  | 0.4 | 0.5 | 2.3 | 0.1 | 0.0 | 0.1 |  | 112 | 103 | 3 | 146 | 166 | 142 |  | Down (2.9) |
| hsa-miR-193a |  | -1.0 | -0.4 | 0.6 | 1.7 | 0.7 | 1.2 |  | 275 | 213 | 58 | 11 | 26 | 33 |  | NC |
| hsa-miR-375 |  | 1.3 | 1.6 | 0.7 | -0.1 | -0.3 | 0.4 |  | 32 | 17 | 47 | 172 | 225 | 101 |  | Down (2.4) |
| hsa-miR-496 |  | 2.1 | 1.2 | -1.2 | 1.0 | -0.1 | 1.1 |  | 9 | 42 | 303 | 46 | 190 | 36 |  | UD |
| hsa-miR-490 |  | -1.6 | -0.3 | 0.7 | 0.8 | 1.3 | 0.6 |  | 304 | 198 | 50 | 67 | 6 | 75 |  | UD |
| hsa-miR-524 |  | 1.1 | 2.2 | 0.4 | -0.6 | -0.1 | -0.4 |  | 44 | 7 | 92 | 237 | 181 | 205 |  | UD |
| hsa-miR-19b |  | 1.3 | 0.8 | 0.5 | 0.1 | 0.3 | 0.3 |  | 31 | 57 | 68 | 152 | 71 | 105 |  | Up (1.2) |
| hsa-miR-525 |  | 0.3 | 0.9 | 0.1 | 1.9 | -0.2 | -0.4 |  | 122 | 53 | 130 | 8 | 200 | 203 |  | UD |
| hsa-miR-515-5p |  | 0.4 | 0.6 | 0.8 | -1.0 | 0.8 | -0.9 |  | 109 | 77 | 38 | 274 | 16 | 265 |  | UD |
| hsa-miR-148a |  | 0.4 | 0.1 | 0.8 | 1.2 | 0.3 | 0.0 |  | 105 | 140 | 36 | 39 | 69 | 158 |  | NC |
| hsa-miR-133b |  | 1.4 | -0.2 | -0.2 | 1.4 | 0.4 | -1.0 |  | 27 | 179 | 211 | 24 | 60 | 266 |  | Down (2.4) |
| hsa-miR-519b |  | 0.4 | 2.1 | 0.0 | 0.0 | 0.4 | 0.0 |  | 111 | 9 | 174 | 156 | 58 | 149 |  | UD |
| hsa-miR-27a |  | 0.2 | 2.4 | -0.1 | 0.3 | 0.1 | -0.1 |  | 135 | 4 | 193 | 115 | 139 | 171 |  | Up (1.1) |
| hsa-miR-95 |  | 0.9 | -0.3 | -0.1 | 1.8 | 0.3 | 1.7 |  | 54 | 205 | 181 | 9 | 93 | 22 |  | Up (0.9) |

Numbers marked with a grey square: Increase in cPARP cleavage (z-score ≥1.5)

Pre-miRs that are in common with the pre-miRs Top-40 ranked (decrease ki67)(Table S5) are in bold

^Δ^numbers in (); log2 fold changes clinical samples (normal colon mucosa vs adenocarcinoma)

NC; no change (Mann Whitney U test p-value < 0.01), UD; undetermined (expressed in ≤ 80% of the samples), NA; not analyzed

**Supplementary Table S5. Rank product Top 40 pre-miRs (decreased Ki67)**

| **Pre-miR ID** |  | **Z-scores** | | | | | |  | **Ranking** | | | | | |  | **Dys-regulation clinical samples** |
| --- | --- | --- | --- | --- | --- | --- | --- | --- | --- | --- | --- | --- | --- | --- | --- | --- |
|  |  | **HCT**  **116** | **HT29** | **SW480** | **DLD1 TR7** | **LS174T TR4** | **Caco2** |  | **HCT**  **116** | **HT29** | **SW480** | **DLD1 TR7** | **LS174T TR4** | **Caco2** |  |  |
| hsa-miR-302a* |  | -1.8 | -1.7 | -2.2 | -1.8 | -0.2 | 0.0 |  | 44 | 14 | 6 | 9 | 139 | 164 |  | UD |
| hsa-miR-369-3p |  | -1.5 | -1.0 | -1.7 | -0.9 | -1.8 | 1.0 |  | 14 | 47 | 15 | 67 | 7 | 271 |  | NC |
| hsa-miR-9 |  | 0.4 | -1.5 | -2.0 | -1.6 | -1.7 | 1.4 |  | 227 | 21 | 8 | 20 | 9 | 298 |  | Down (2.0)^Δ^ |
| hsa-miR-23b |  | 0.2 | -1.9 | -0.9 | -2.0 | 0.0 | 0.2 |  | 190 | 9 | 52 | 3 | 175 | 201 |  | Down (1.7) |
| hsa-miR-455 |  | 0.2 | -2.5 | -0.6 | -1.3 | -1.0 | 0.3 |  | 203 | 3 | 75 | 44 | 38 | 213 |  | Up (0.6) |
| **hsa-miR-373*** |  | 0.2 | -1.4 | -1.6 | 1.6 | -1.8 | 0.0 |  | 196 | 23 | 16 | 304 | 4 | 165 |  | UD |
| hsa-miR-342 |  | -1.1 | -1.2 | -1.0 | -1.8 | 0.6 | 2.2 |  | 34 | 31 | 42 | 8 | 254 | 313 |  | UD |
| hsa-miR-302a |  | -0.8 | -1.6 | -2.3 | 0.2 | -0.1 | -1.8 |  | 58 | 16 | 4 | 175 | 151 | 12 |  | UD |
| hsa-miR-181b |  | -0.3 | -1.7 | -1.5 | -1.4 | -0.5 | 0.0 |  | 127 | 15 | 18 | 40 | 77 | 174 |  | NA |
| hsa-miR-516-5p |  | -0.5 | -1.8 | -1.3 | -1.8 | 1.3 | -0.8 |  | 113 | 12 | 27 | 10 | 292 | 68 |  | UD |
| hsa-miR-514 |  | -0.7 | -0.9 | -1.3 | -1.3 | -1.2 | 1.6 |  | 72 | 62 | 29 | 43 | 21 | 303 |  | NA |
| hsa-miR-520a |  | -1.8 | -1.3 | -0.4 | -0.4 | -0.8 | 0.9 |  | 7 | 28 | 106 | 118 | 56 | 256 |  | UD |
| hsa-miR-103 |  | -0.8 | -1.8 | -0.3 | -1.6 | -0.4 | 1.2 |  | 60 | 10 | 116 | 24 | 92 | 282 |  | NC |
| hsa-miR-99b |  | -0.1 | -1.8 | -0.9 | -1.6 | -0.2 | 0.9 |  | 162 | 11 | 46 | 16 | 137 | 260 |  | NC |
| hsa-miR-196b |  | -1.1 | 0.3 | -0.4 | -1.6 | -1.5 | 1.9 |  | 37 | 203 | 112 | 19 | 12 | 308 |  | NC |
| hsa-miR-424 |  | -1.3 | -0.2 | 0.9 | -1.1 | -1.8 | 1.0 |  | 22 | 126 | 269 | 53 | 5 | 264 |  | Up (2.0) |
| hsa-miR-367 |  | -0.9 | -0.3 | -1.2 | -1.5 | -1.0 | 1.0 |  | 54 | 111 | 34 | 30 | 33 | 267 |  | UD |
| hsa-miR-409-5p |  | -0.9 | -1.1 | -2.1 | -0.8 | 1.1 | 1.9 |  | 55 | 34 | 7 | 74 | 283 | 309 |  | NC |
| hsa-miR-182* |  | -1.2 | -1.5 | 0.3 | -1.3 | -0.8 | -0.3 |  | 26 | 19 | 209 | 45 | 60 | 127 |  | UD |
| hsa-miR-302b* |  | -1.1 | -0.8 | -1.5 | 0.6 | -0.9 | -0.5 |  | 33 | 65 | 17 | 229 | 42 | 89 |  | UD |
| hsa-miR-323 |  | -0.6 | 0.1 | 0.2 | -1.2 | -1.9 | -0.3 |  | 85 | 161 | 193 | 49 | 3 | 126 |  | NA |
| hsa-miR-200a* |  | 2.4 | -2.8 | 0.6 | -1.5 | -0.3 | -0.8 |  | 311 | 2 | 236 | 25 | 109 | 65 |  | NC |
| hsa-miR-181a |  | -0.5 | 0.0 | -0.2 | 0.2 | -2.0 | -2.4 |  | 112 | 150 | 135 | 180 | 1 | 2 |  | NC |
| hsa-miR-519e |  | -1.2 | -1.3 | -0.1 | -0.6 | -0.8 | -0.9 |  | 24 | 25 | 142 | 92 | 53 | 59 |  | UD |
| hsa-miR-99a |  | -3.9 | -0.3 | 0.2 | -0.3 | -0.1 | 0.2 |  | 1 | 120 | 186 | 127 | 156 | 204 |  | NC |
| **hsa-miR-183** |  | -1.6 | -1.5 | 1.2 | -0.6 | -0.6 | 2.6 |  | 12 | 20 | 290 | 94 | 70 | 316 |  | Up (1.3) |
| **hsa-miR-452*** |  | -1.7 | -1.5 | -0.7 | 0.1 | 0.7 | 1.0 |  | 9 | 17 | 71 | 167 | 264 | 269 |  | NC |
| hsa-miR-194 |  | 0.3 | 0.4 | -2.9 | -0.3 | -0.5 | -1.0 |  | 213 | 215 | 1 | 124 | 87 | 53 |  | Down (0.7) |
| hsa-miR-202 |  | -0.7 | 0.1 | -0.1 | -2.6 | 1.0 | -0.3 |  | 74 | 164 | 149 | 1 | 277 | 115 |  | NC |
| hsa-miR-17-5p |  | 0.7 | -2.1 | -0.6 | -0.5 | -1.0 | 0.5 |  | 255 | 7 | 79 | 103 | 37 | 230 |  | Up (0.9) |
| hsa-miR-325 |  | 0.6 | -3.3 | -1.0 | 0.8 | 0.4 | 0.9 |  | 246 | 1 | 41 | 248 | 227 | 261 |  | UD |
| hsa-miR-520d* |  | 0.4 | -1.1 | -0.2 | -2.0 | -0.4 | -0.2 |  | 222 | 40 | 132 | 5 | 97 | 131 |  | NA |
| **hsa-let-7i** |  | -1.4 | 0.0 | -0.7 | -1.6 | -0.1 | 0.3 |  | 19 | 146 | 64 | 21 | 164 | 215 |  | NA |
| hsa-miR-214 |  | -1.2 | -1.1 | -0.6 | -0.7 | -0.5 | -0.4 |  | 30 | 37 | 82 | 86 | 80 | 104 |  | NC |
| hsa-miR-138 |  | 0.0 | -1.7 | -0.8 | -1.4 | -0.1 | -0.2 |  | 172 | 13 | 56 | 34 | 154 | 140 |  | Down (2.3) |
| **hsa-miR-185** |  | -0.8 | -1.0 | -2.2 | 0.0 | 1.1 | 2.2 |  | 63 | 52 | 5 | 152 | 282 | 312 |  | NC |
| hsa-miR-129 |  | -0.5 | -1.9 | -0.2 | -0.7 | -0.5 | 1.0 |  | 105 | 8 | 133 | 83 | 76 | 277 |  | UD |
| hsa-let-7d |  | -1.7 | -1.1 | 0.8 | -1.4 | 0.4 | 1.0 |  | 10 | 38 | 255 | 35 | 231 | 270 |  | Down (0.9) |
| **hsa-miR-369-5p** |  | -1.3 | -0.1 | -1.7 | -0.8 | 0.6 | -1.5 |  | 23 | 131 | 13 | 78 | 257 | 23 |  | NC |
| **hsa-let-7b** |  | -0.7 | -0.3 | 1.5 | 0.0 | -2.0 | 1.0 |  | 77 | 121 | 297 | 153 | 2 | 268 |  | NC |

Numbers marked with grey square: Decrease in decreased Ki67 (z-score ≤ -1.5)

Pre-miRs that are in common with the pre-miRs Top-40 ranked (increased cPARP cleavage)(Table S4) are in bold

^Δ^numbers in (); log2 fold changes clinical samples (normal colon mucosa vs adenocarcinoma)

NC; no change (Mann Whitney U test p-value < 0.01), UD; undetermined (expressed in ≤ 80% of the samples), NA; not analyzed
